# Genomic profiling of stage II and III colon cancers reveals *APC* mutations to be associated with survival in stage III colon cancer patients

**DOI:** 10.18632/oncotarget.12510

**Published:** 2016-10-06

**Authors:** Evert van den Broek, Oscar Krijgsman, Daoud Sie, Marianne Tijssen, Sandra Mongera, Mark A. van de Wiel, Eric J. Th. Belt, Sjoerd H. den Uil, Herman Bril, Hein B.A.C. Stockmann, Bauke Ylstra, Beatriz Carvalho, Gerrit A. Meijer, Remond J.A. Fijneman

**Affiliations:** ^1^ Department of Pathology, VU University Medical Center, Amsterdam, The Netherlands; ^2^ Department of Epidemiology and Biostatistics, VU University Medical Center, Amsterdam, The Netherlands; ^3^ Department of Mathematics, VU University, Amsterdam, The Netherlands; ^4^ Department of Surgery, VU University, Amsterdam, The Netherlands; ^5^ Department of Pathology, Spaarne Gasthuis, Haarlem, The Netherlands; ^6^ Department of Pathology, Netherlands Cancer Institute, Amsterdam, The Netherlands

**Keywords:** colon cancer, copy number aberrations, APC, structural variants, disease recurrence

## Abstract

Tumor profiling of DNA alterations, i.e. gene point mutations, somatic copy number aberrations (CNAs) and structural variants (SVs), improves insight into the molecular pathology of cancer and clinical outcome. Here, associations between genomic aberrations and disease recurrence in stage II and III colon cancers were investigated. A series of 114 stage II and III microsatellite stable colon cancer samples were analyzed by high-resolution array-comparative genomic hybridization (array-CGH) to detect CNAs and CNA-associated chromosomal breakpoints (SVs). For 60 of these samples mutation status of *APC, TP53, KRAS, PIK3CA, FBXW7, SMAD4*, *BRAF* and *NRAS* was determined using targeted massive parallel sequencing. Loss of chromosome 18q12.1-18q12.2 occurred more frequently in tumors that relapsed than in relapse-free tumors (*p* < 0.001; FDR = 0.13). In total, 267 genes were recurrently affected by SVs (FDR < 0.1). CNAs and SVs were not associated with disease-free survival (DFS). Mutations in *APC* and *TP53* were associated with increased CNAs. *APC* mutations were associated with poor prognosis in (5-fluorouracil treated) stage III colon cancers (*p* = 0.005; HR = 4.1), an effect that was further enhanced by mutations in MAPK pathway (*KRAS, NRAS, BRAF*) genes. We conclude that among multiple genomic alterations in CRC, strongest associations with clinical outcome were observed for common mutations in *APC*.

## INTRODUCTION

Colorectal cancer (CRC) is a major health care problem and is the second leading cause of cancer-related deaths in the Western world. The worldwide incidence of CRC is over 1.3 million with a mortality rate of about 50% [1]. In current clinical practice, cancer patients are classified according to tumor-node-metastasis (TNM) staging, which is primarily based on histopathological features of the tumor. Adjuvant chemotherapy is not recommended for treatment of stage II colon cancer, except for patients with high-risk features [2]. However, approximately 20% of stage II colon cancers will develop disease recurrence after resection of the primary tumor [3]. In contrast, stage III colon cancer patients generally do receive adjuvant chemotherapy. Nevertheless, approximately 40% of these patients will develop a relapse [4]. Therefore, stage II and III colon cancer patients are a clinically relevant group for risk stratification of disease recurrence.

Cancer is a genetic disease that arises by the accumulation of somatic DNA alterations, a process that is accelerated by genome instability. These mutations include non-synonymous point mutations, somatic chromosomal copy number aberrations (CNAs) and structural variants (SVs) [5]. The resulting activation of oncogenes and inactivation of tumor suppressor genes enables tumors to progress. Therefore, the genetic make-up of the tumor is the blueprint of its biological and clinical behavior, and characterization of these irreversible DNA aberrations is expected to facilitate patient stratification [6]. Based on the mechanisms that cause genome instability in CRC, two molecular classes of CRC are currently distinguished, *i.e.* microsatellite instability (MSI) and chromosomal instability. Most microsatellite stable (MSS) tumors, which comprise approximately 85% of all CRCs, exhibit chromosomal instability resulting in gains and losses of relatively large chromosomal segments. Clinically, stage II MSI tumors have a favorable prognosis compared to stage II MSS tumors, while the opposite is the case for tumors with distant metastases [7, 8]. Further classification of MSS CRCs into clinically relevant subtypes was recently achieved by an international consortium using gene expression analysis, which yielded the ‘consensus molecular subtypes’ [9]. However, attempts to classify these RNA-based MSS CRC subtypes also by DNA mutation analysis were not successful. At present, strong DNA-based molecular indicators for tumor relapse of MSS stage II and stage III colon cancers are lacking. Comprehensive genomic profiling of MSS colon cancers may gain insight in underlying molecular pathology that contributes to disease recurrence and thereby improve patient stratification for treatment with adjuvant therapy.

## RESULTS

### Loss of 18q12.1-18q12.2 was associated with tumor relapse

**Figure 1:**
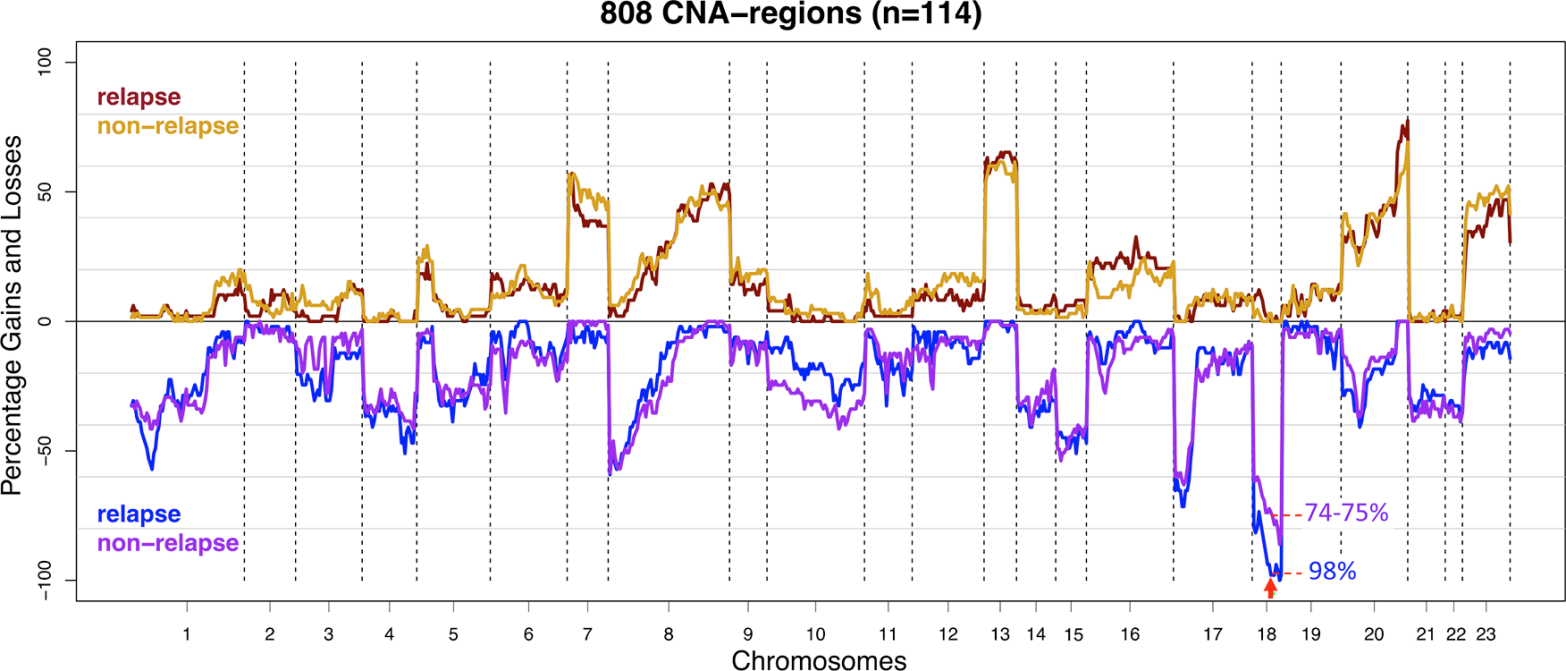
Frequency plot of copy number gains and losses of stage II and III colon cancer samples (*n* = 114) stratified for disease recurrence The CNA frequencies of cases that developed disease recurrence (*n* = 49) were represented by red for gains and blue for losses. CNA frequencies of relapse-free cases (*n* = 65) were represented by orange and purple for gains and losses respectively. The X-axis depicts chromosomes 1-22 and X (numbered 23) with chromosome boundaries indicated by vertical dotted lines. The Y-axis depicts the percentage of observed copy number aberrations: gains (above zero line) and losses (below zero line). Copy number loss of chromosome 18q12.1 - 18q12.2 (marked with red arrow) was more frequently observed in samples from tumors that developed disease recurrence (*p* < 0.001; FDR = 0.13).

A total of 57 stage II and 57 stage III primary colon cancers were selected for array-CGH analysis to determine DNA copy number profiles. Of these, 22 stage II (39%) and 27 stage III (47%) colon cancers had a disease recurrence (Table 1). Previously reported common CNAs characterizing colon cancers, *i.e.* loss of chromosomes 1p, 4, 8p, 17p and 18 as well as gain of chromosomes 7, 8q, 13q and 20q [10–13] were concordantly observed in this series of samples (Figure 1). Unsupervised hierarchical cluster analysis revealed no association of patterns of CNAs and tumor stage or relapse (Supplementary Figure S1). Next, a supervised analysis of CNAs showed loss of two contiguous regions located on chromosome 18q12.1 - 18q12.2 in 98% of tumors that relapsed versus 74–75% in relapse-free tumors (*p* < 0.001; FDR = 0.13; Figure 1; Supplementary Table S4). Further evaluation of putative effects of CNAs on disease-free survival (DFS) revealed that copy number loss of these regions was not significantly associated with poor survival (*p* < 0.005; FDR = 0.43; Supplementary Table S4; Supplementary Figure S2). The comparison of CNAs in stage II to stage III colon cancers also did not reveal any significant differences (Supplementary Table S4).

**Table 1:** Baseline clinicopathological characteristics of 114 MSS stage II and III colon cancer patients

		Overall (*n* = 114)	Stage II (*n*= 57)	Stage III (*n* = 57)
**Sex**	Male	67 (58.8)	31 (54.4)	36 (63.2)
	Female	47 (41.2)	26 (45.6)	21 (36.8)
**Age (years)**	Mean (s.d.)	69.2 (11.6)	72,0 (12.4)	66.4 (10.1)
	Median (range)	70.1 (28.5–91.3)	74.4 (28.5–91.3)	66.6 (40.9–83.3)
**Tumor location**	Right sided	46 (40.4)	19 (33.3)	27 (47.4)
	Left sided	68 (59.6)	38 (66.7)	30 (52.6)
**Tumor size (mm)**	Mean (s.d.)	36.8 (16.3)	39.3 (19.4)	34.4 (12.4)
**Tumor stage**	T1	1 (0.9)	0 (0.0)	1 (1.8)
	T2	7 (6.1)	0 (0.0)	7 (12.3)
	T3	96 (84.2)	53 (93.0)	43 (75.4)
	T4	10 (8.8)	4 (7.0)	6 (10.5)
**Nodal stage**	N0	57 (50.0)	57 (100.0)	0 (0.0)
	N1	36 (31.6)	0 (0.0)	36 (63.2)
	N2	21 (18.4)	0 (0.0)	21 (36.8)
**No. of nodes examined**	Mean (s.d.)	9.0 (4.1)	7.9 (3.6)	10.1 (4.4)
**Histological grade**	Well	8 (7.02)	6 (10.5)	2 (3.5)
	Moderate	99 (86.8)	49 (86.0)	50 (87.7)
	Poor	7 (6.1)	2 (3.5)	5 (8.8)
**Mucinous**	No	96 (84.2)	45 (78.9)	51 (89.5)
**differentiation**	Yes	18 (15.8)	12 (21.1)	6 (10.5)
**Ulceration**	Absent	21 (18.4)	12 (21.1)	9 (15.8)
	Present	93 (81.6)	45 (78.9)	48 (84.2)
**Angioinvasion**	Absent	83 (72.8)	49 (86.0)	34 (59.6)
	Present	31 (27.2)	8 (14.0)	23 (40.4)
**Adjuvant therapy**	No	56 (49.1)	55 (96.5)	1 (1.8)
	Yes*	58 (50.9)	2 (3.5)	56 (98.2)
**Recurrent Disease**	No	65 (57.0)	35 (61.4)	30 (52.6)
	Yes	49 (43.0)	22 (38.6)	27 (47.4)
**Follow-up (months)**	Median (range)	57.3 (4.3–129.2)	61.8 (11.5–129.2)	53,6 (4.3–127.4)

Values in parentheses are percentages unless stated otherwise.

* Adjuvant chemotherapy: 5-fluorouracil and leucovorin (5-FU/LV) mono therapy.

### Identification of 267 CNA-associated recurrent breakpoint genes

**Figure 2:**
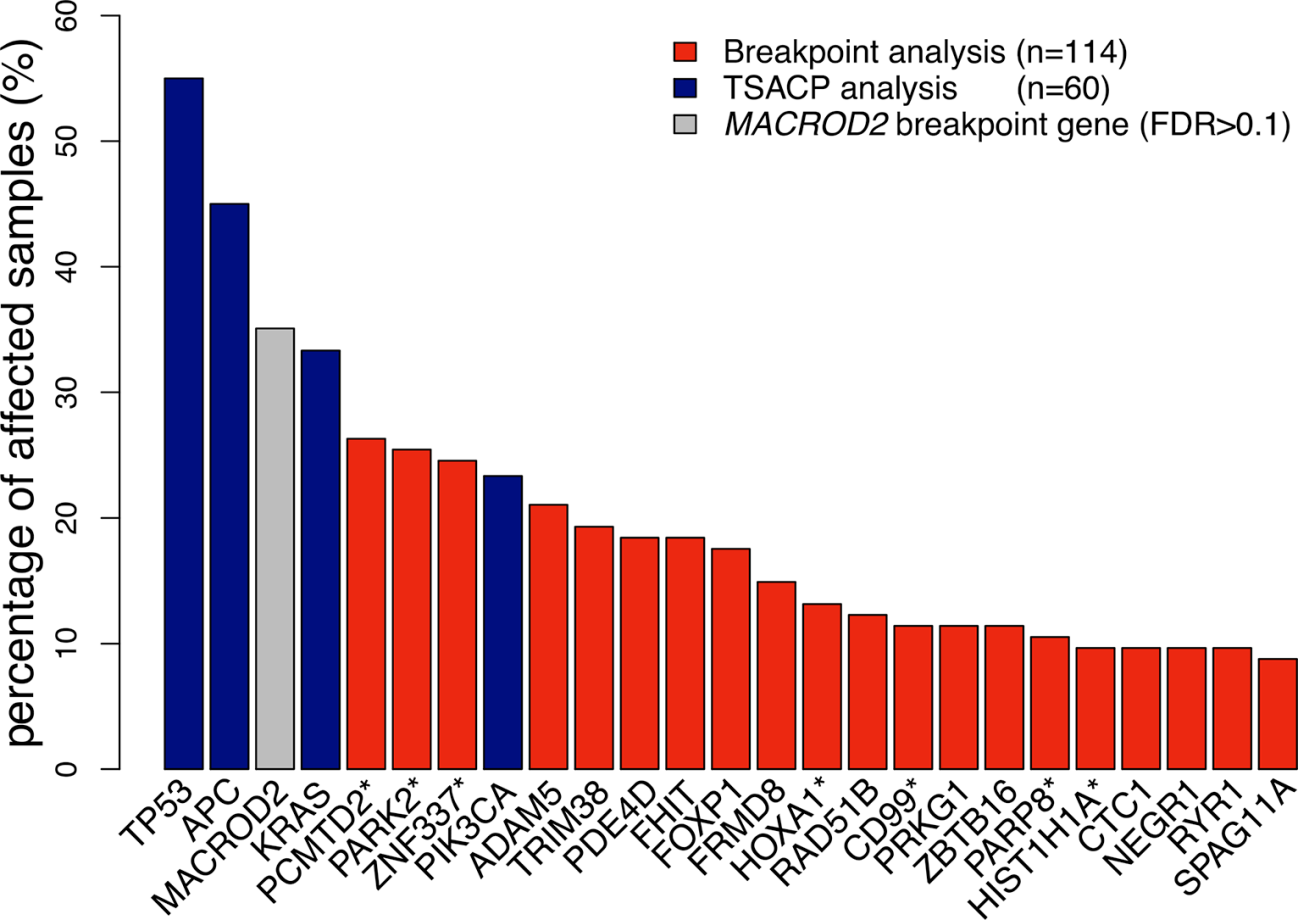
Gene breakpoint and gene mutation frequencies of the 25 most frequently affected genes in this series of colon cancers Gene breakpoint frequencies (red bars) were based on the analysis of 114 colon cancer samples and gene mutation frequencies (blue bars) on the analysis of 60 samples. *MACROD2* (grey bar) did not reach the level of significance in this series of samples (FDR = 0.17). Genes marked with a “*” indicate a pool of genes that share probe(s) associated with chromosomal breakpoints: the PCMTD2* pool also includes *LINC00266-1*; PARK2* also includes PACRG; ZNF337* also includes *NCOR1P1*, *FAM182A*, *FAM182B*, *FRG1B*, *MIR663A* and *MLLT10P1*; HOXA1* also includes *HOXA2*; CD99* also includes XG; PARP8* also includes *EMB*; HIST1H1A* also includes HIST1H3A. (See also Supplementary Table S5) The frequency of affected samples in the pool of genes was determined by the cumulative mutation frequency of pooled genes.

Array-CGH profiles also allow detection of CNA-associated chromosomal breakpoints, which indicate genomic locations that are affected by double strand breaks [14]. In the present series of 114 colon cancer samples a total of 314 non-random chromosomal breakpoint locations were identified by cohort-based statistical analysis (FDR < 0.1; Supplementary Table S1 and Supplementary Figure S3). A total of 267 genes were detected that were recurrently affected by CNA-associated breakpoints (FDR < 0.1; Supplementary Table S2 and Supplementary Figure S3). These are further referred to as recurrent breakpoint genes. Compared to a previous study in which 748 recurrent breakpoint genes were identified in a series of 352 advanced CRC samples [14], there is a significant overlap of 168 genes (63%). In both studies *MACROD2* is the gene that is most frequently affected by chromosomal breaks, *i.e.* in 35% of stage II and III colon cancer samples in the present dataset and in 41% of advanced CRCs [14]. However, *MACROD2* did not reach the level of statistical significance in the present dataset (FDR = 0.17). Nevertheless, we did include *MACROD2* for further analyses because of its highly significant identification as a recurrent breakpoint gene in the advanced CRC series (FDR = 3.6E-11) [14]. Besides *MACROD2*, 23 recurrent breakpoint genes (FDR < 0.1) were detected at a relatively high prevalence, affecting more than 10% of colon cancer samples (Figure 2; Supplementary Figure S3; Supplementary Table S2). None of these 24 highly prevalent breakpoint genes were significantly associated with DFS in a univariate analysis when stage II and III colon cancers were combined or evaluated separately (log-rank test; data not shown).

### *APC* mutations are associated with poor DFS in stage III colon cancer

**Figure 3:**
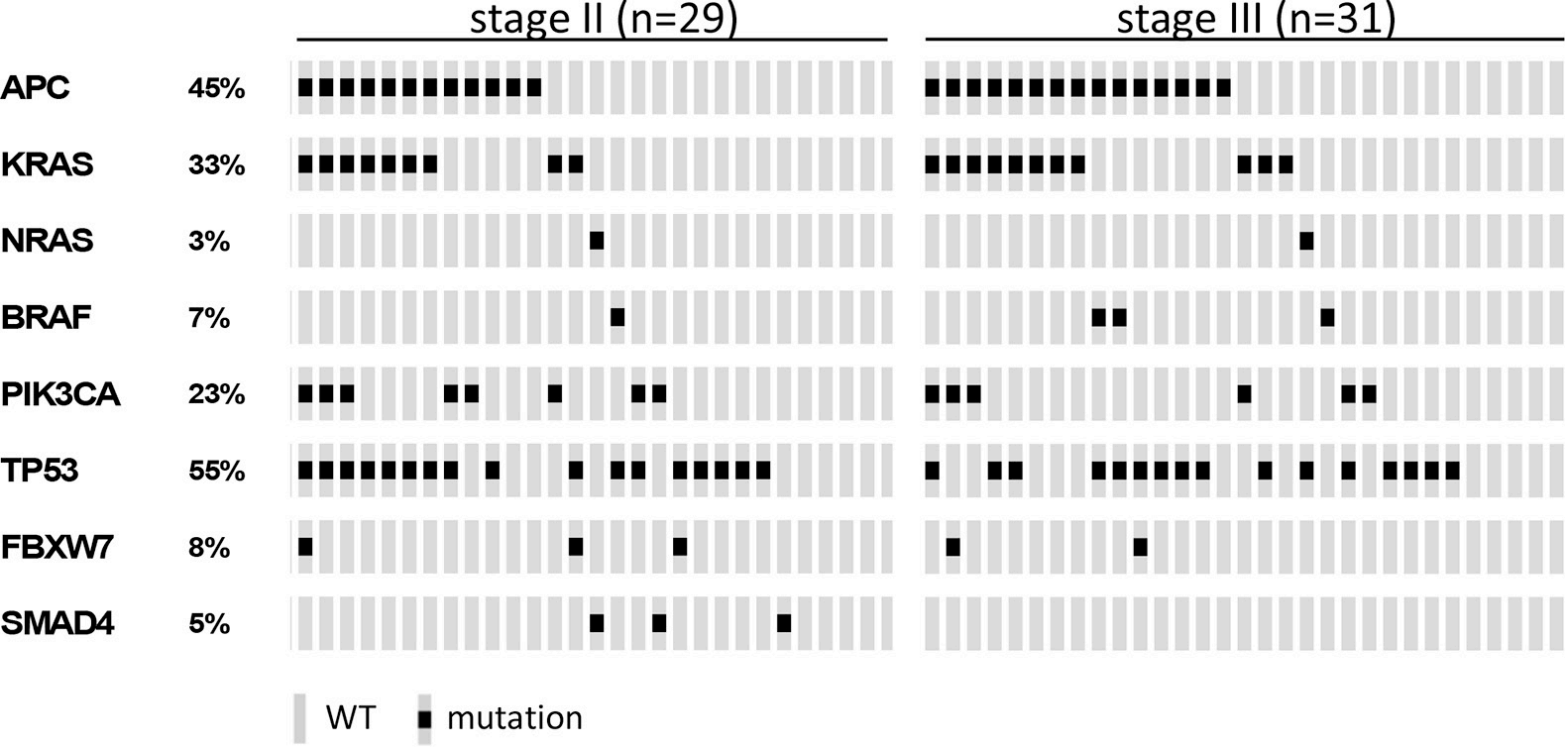
Oncoprint visualizing the gene mutation status of *APC*, *KRAS*, *NRAS*, *BRAF*, *PIK3CA*, *TP53*, *FBXW7* and *SMAD4* assessed by TruSeq Amplicon Cancer Panel TSACP analysis for stage II (*n* = 29) and stage III (*n* = 31) colon cancers The rows indicate the gene mutation status of the 60 samples (grey bars) and the black spots depict mutations.

**Figure 4:**
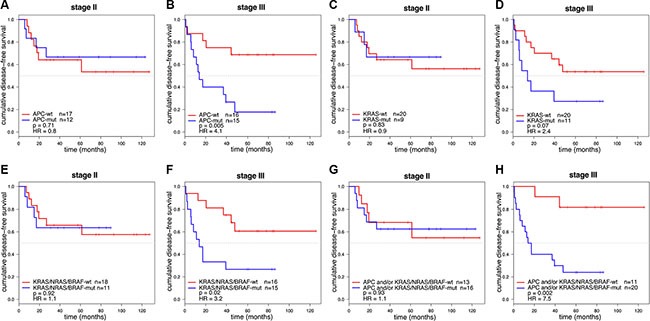
Kaplan-Meier curves of DFS for stage II and stage III colon cancer patients (*n* = 60) DFS curves were stratified for *APC* mutation in 29 stage II (**A**) and 31 stage III (**B**) colon cancer patients. Stage III colon cancers with an *APC* mutation were associated with a poor prognosis (*p* = 0.005; HR = 4.1). DFS curves stratified for *KRAS* mutation in stage II (**C**) and stage III (**D**) colon cancers showed that *KRAS* mutations were associated with a worse DFS in stage III (*p* = 0.07; HR = 2.4). DFS curves stratified for mutations in the *KRAS/NRAS/BRAF* MAPK pathway genes in stage II (**E**) and stage III (**F**) colon cancers showed that tumors with a mutation in one of the MAPK pathway genes were associated with a poor prognosis in stage III colon cancers (*p* = 0.02; HR = 3.2). DFS curve stratified for patients having a mutation in *APC* and/or MAPK pathway genes (*KRAS*, *BRAF* or *NRAS*) for stage II (**G**) and stage III (**H**) colon cancers showed that tumors with a mutation in *APC* and/or one of the MAPK pathway genes were associated with a poor prognosis in stage III colon cancers (*p* = 0.002; HR = 7.5).

For 29 stage II and 31 stage III colon cancer samples, mutation analysis of *APC*, *TP53*, *KRAS*, *PIK3CA*, *FBXW7*, *SMAD4*, *BRAF* and *NRAS* passed quality control (Supplementary Table S3). Of these, 11 stage II (38%) and 17 stage III (55%) colon cancers had developed disease recurrence (Supplementary Table S3). The observed mutation frequencies are in concordance with published data (Figure 3 and Supplementary Table S6 and S7), taking into account that the TruSeq Amplicon Cancer Panel (TSACP) targeted massive parallel sequencing approach did not cover the complete *APC* gene [10, 15]. In the present study, *APC* mutations were identified in 45% of colon cancers, while the frequency of *APC* mutations in MSS CRC samples published by The Cancer Genome Atlas (*n* = 195) [10] would have been 51% when restricted to regions of the gene that are covered by the TSACP. No associations were found between gene mutation status and DFS in stage II colon cancers (Figure 4; Supplementary Figure S4), while stage III patients carrying an *APC* mutation had a worse DFS compared to patients without *APC* mutation (*p* = 0.005; HR=4.1; Figure 4B). In addition, stage III patients carrying a *KRAS* mutation tended to have a worse DFS compared to patients without *KRAS* mutation (*p* = 0.07; HR=2.4; Figure 4D). In accordance with data from The Cancer Genome Atlas, mutations in *KRAS*, *BRAF* and *NRAS* are nearly mutually exclusive (Figure 3) because these genes interact in the MAPK pathway [10, 16]. When mutations in these MAPK pathway genes were combined, they were associated with poor DFS in stage III colon cancer patients (*p* =0.02; HR=3.2; Figure 4F). For stage II colon cancers no significant association was observed between MAPK pathway gene mutation status and DFS (Figure 4E).

Mutations in *APC* and *KRAS* were frequently co-occurring in this series of 60 MSS colon cancer samples (Fisher-Exact: *p* = 0.002; Figure 3). This observation was validated in an independent series of 180 MSS advanced CRCs that was similarly analyzed for these gene mutations (Fisher-Exact: *p* = 0.03; Supplementary Figure S5) [14]. The difference in DFS of stage III colon cancer patients that harbored a mutation in either *APC* and/or a mutation in one of the *KRAS*, *BRAF*, *NRAS* genes from the MAPK pathway compared to patients that were wild-type (WT) for these genes was even more pronounced than for *APC* mutation status alone (*p* = 0.002; HR =7 .5; Figure 4H), while such an association was not observed in stage II patients (Figure 4G).

### *APC* and *TP53* gene mutations are associated with a high CNA-score

**Figure 5:**
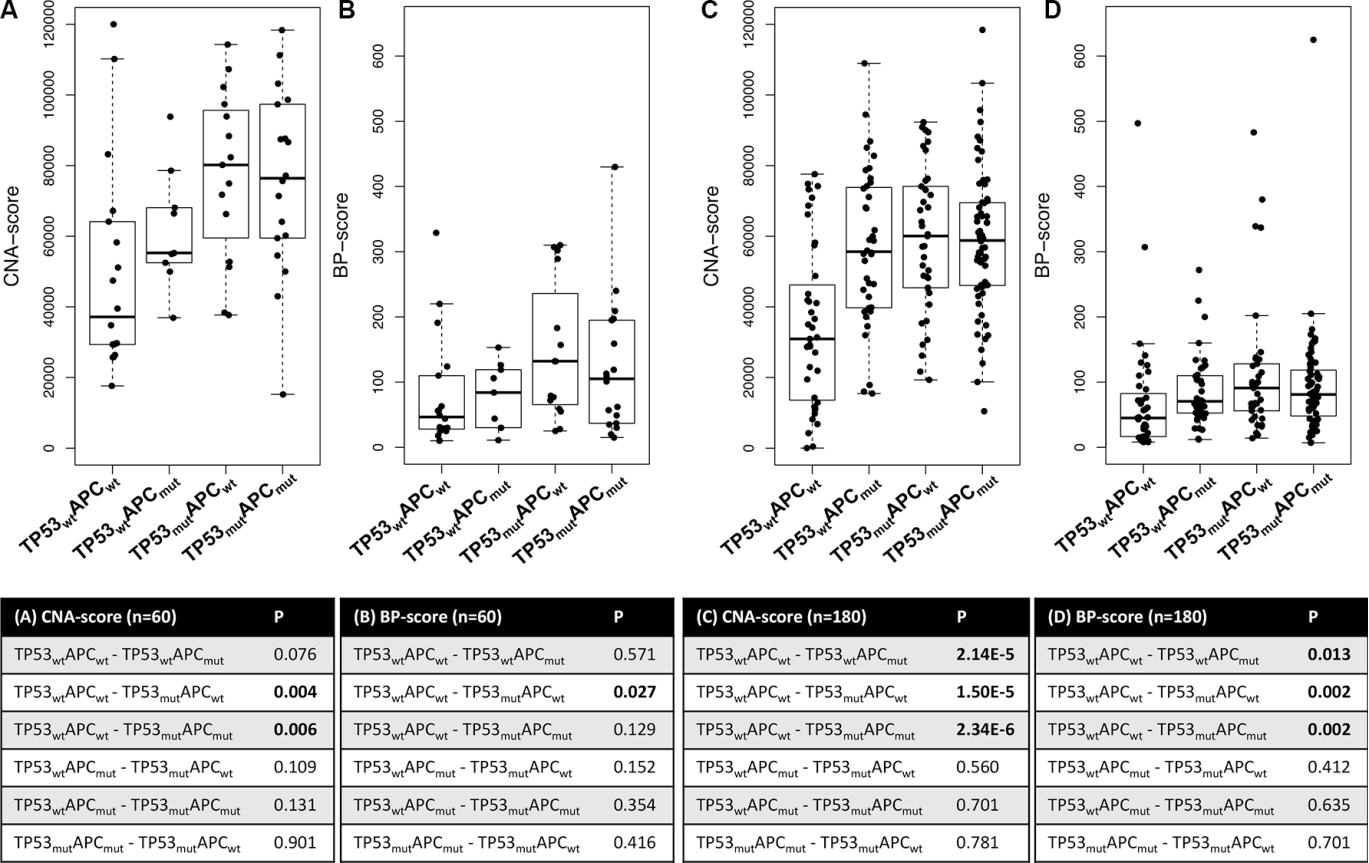
Boxplots of CNA- and BP-scores for four subgroups of CRC samples stratified for *APC* and *TP53* mutation status (**A**) CNA-scores and (**B**) BP-scores for stage II and III MSS colon cancers (*n* = 60). Statistical analyses using two-sided Mann-Whitney U tests revealed that *TP53* mutation was associated with increased CNAs (*p* = 0.004) and BPs (*p* = 0.03). (**C**) CNA-scores and (**D**) BP-scores for advanced MSS CRC samples [13]. Both *APC* and *TP53* mutations were associated with increased CNA- and BP-scores.

The degree of chromosomal instability for each individual tumor can be indicated by the number of array-CGH probes across the genome that is gained or lost (CNA-score) and by the extent of chromosome fragmentation represented by the number of chromosomal breakpoint locations (BP-score). TP53 is known as a gatekeeper of the genome, maintaining genome integrity [17, 18]. A strong positive association between TP53 mutation and the degree of chromosomal instability was observed for CNA- and BP-scores (two-sided Mann-Whitney *U* test; *p* < 0.001 and *p* = 0.02, respectively). Also APC has been implicated to play a role in chromosomal instability [19–21]. Neither CNA- nor BP-scores were associated with APCmutation status by univariate analysis (*p* = 0.2 and *p* = 0.9, respectively). Considering the substantial effect of TP53 mutations on CNA- and BP-scores, the effects of APC mutations were also examined in samples that lacked mutations in TP53. Comparison of APC mutant TP53-WT versus TP53-APC double WT cancers tended to show an association of APC mutations with CNA-score (*p* = 0.08) while no association was observed with BP-score (*p* = 0.6) (Figure 5A–5B). To validate association of APC mutations with CNA-score, this analysis was repeated in the same independent series of MSS advanced CRCs (*n* = 180) mentioned above [14]. In addition to confirmation of the strong effect of TP53 mutations in APC-WT cancers on CNA- and BP-scores (*p* =1.5E-5 and *p* = 0.002, respectively) (Figure 5C–5D), it also revealed that APC mutations in TP53-WT cancers were associated with increased CNA- and BP-scores compared to TP53-APC double WT cancers (*p* = 2.1E-5 and *p* = 0.01, respectively) (Figure 5C–5D). Cancers in which both TP53 and APC were mutated did not show additional effects on CNA- and BP-scores compared to cancers with mutations in either one of these genes (Figure 5).

## DISCUSSION

Studies that comprehensively investigated the prognostic value of common gene mutations, CNAs and SVs in stage II and III colon cancer are sparse. The present study aimed to gain insight into somatic DNA aberrations that contribute to disease recurrence of stage II and III MSS colon cancers by genomic profiling for gene mutations, CNAs and CNA-associated SVs. The main finding of this study is that *APC* mutations, as could be detected by coverage of the TSACP analysis, were associated with a poor DFS in stage III MSS colon cancer patients (*p* = 0.005; HR = 4.1; Figure 4B). Moreover, the association with survival was even stronger when *APC* mutation status was combined with mutation status of the MAPK pathway genes *KRAS*, *BRAF* and *NRAS* (*p* = 0.002; HR = 7.5; Figure 4H). Similar to tumors with a *TP53* mutation, the tumors with an *APC* mutation also showed increased CNA- and BP-scores indicative for a supportive role of *APC* mutations in chromosomal instability. Moreover, we demonstrated that loss of chromosome 18q12.1 - 18q12.2 was associated with disease recurrence (*p* < 0.001; FDR = 0.13; Figure 1). Taken together, mutations in *APC*, *TP53*, *KRAS* and loss of chromosome 18q, *i.e.* genomic aberrations that are highly prevalent and well-known from the colon tumor progression model [22, 23], were associated with disease deterioration in this series of MSS colon cancers.

The functional contribution of *APC* mutations to progression of MSS stage III colon cancers is currently not fully understood. The aggressiveness and subsequent worse outcome of MSS stage III tumors with *APC* mutations may be driven by WNT activity, since *APC* is a key negative regulator of the WNT canonical pathway [24]. The observations that mutations in *APC* and *KRAS* are co-occurrent and that the impact on DFS was further enhanced in an analysis that combined *APC* mutations with mutations in *KRAS*, *BRAF* and *NRAS* MAPK pathway genes, suggesting a synergistic effect of coinciding activation of WNT and MAPK signaling pathways. Several studies provided evidence that supports this hypothesis. It has been demonstrated that oncogenic mutations in *KRAS* facilitate the nuclear translocation of β-catenin [25], which is accumulated in the cytoplasm as a result of decreased destruction complex activity by loss of *APC* function. Furthermore, enhanced WNT activity was reported in cells with a *KRAS* mutation in addition to *APC* loss [26]. In mice, presence of a *KRAS* mutation in an *APC* mutant background was also associated with reduced survival due to accelerated growth of intestinal tumors [26, 27]. Interestingly, data from the Genomics of Drug Sensitivity in Cancer (GDSC) initiative [28] indicate that *APC* mutations confer increased sensitivity to the MAPK pathway MEK1/2 inhibitor PD-0325901 (www.cancerRxgene.org, release version 5.0). At the same time, *APC* mutations confer increased resistance to the PI3K pathway inhibitors Temsirolimus, NVP-BEZ235 and AZD6482, targeting PI3K and mTOR. As both the MAPK and PI3K pathways can be activated through EGFR tyrosine kinase receptor signaling [29], our data suggest that tumors with *APC* mutations become relatively more dependent on signaling through the MAPK pathway.

While mutations in the *APC* tumor suppressor gene were associated with a worse clinical outcome in stage III MSS colon cancer patients, this association was not encountered in stage II disease. Since none of these stage II and all of the stage III colon cancer patients received 5-fluorouracil (5-FU)-based adjuvant chemotherapy, it is not possible to discern whether *APC* mutations confer resistance to adjuvant chemotherapy or whether this difference in prognosis is due to effects of *APC* mutations on biology of cancer cells that metastasize to the lymph nodes. Both scenarios are supported by data from literature. With respect to 5-FU treatment, it has been described that CRC patients with *APC* mutations do not benefit from 5-FU therapy [30], possibly due to interaction of 5-FU with the *APC* DNA repair inhibitory domain [31]. Reduced sensitivity to 5-FU was also demonstrated in *APC* mutant colorectal cancer (CRC) cell lines [32]. Inhibition of DNA replication checkpoint by a Chk1 inhibitor could increase sensitivity of *APC*-mutant colon cancer cells [32]. With respect to tumor biology, we previously investigated the expression of MGL ligand and demonstrated strong association with DFS in stage III, but not stage II, colon cancers [33]. High expression of MGL ligand was correlated to *BRAF* mutations and altered glycosylation, which was thought to subsequently increase immunosuppression by metastasizing cancer cells [33]. We hypothesize that in addition to *BRAF* mutations also other mutations in the MAPK pathway, possibly in synergy with *APC* mutations, induce evasion from the immune system.

We also demonstrated a significant association of *APC* mutations with increased CNA- and BP-scores. It has been established that the malignant transformation of the majority of MSS colon tumors is accompanied by chromosomal instability [11, 34]. It is well-established that *TP53* maintains the integrity of the genome, and that *TP53* deficiency contributes to chromosomal instability [35]. The role of *APC* herein is less established, although some studies have described that loss of *APC* function was associated with aneuploidy [19–21, 36, 37]. While the mechanism of *APC* involvement in chromosomal instability remains not fully understood, it has been reported that *APC* can regulate kinetochore microtubule attachment during mitosis [38, 39]. Inactivated *APC* may disrupt chromosome segregation followed by an increase in mitotic abnormalities associated with chromosomal instability [18, 36, 38].

Besides effects of gene mutations, also the effects of relatively large chromosomal CNA segments have been investigated in relation to tumor relapse. The observation that loss of the relatively large segment on chromosome 18 (18q12.1 - 18q12.2) was more frequently detected in colon cancers that developed disease recurrence (FDR = 0.13), suggests that genes encoded at this region may be important for tumor metastasis. One of the genes annotated on chromosome 18q12.1 - 18q12.2 is *ZNF24*. This transcription factor negatively regulates expression of VEGF, which is a key regulator involved in angiogenesis [40, 41]. Compared to normal tissue, *ZNF24* is often down-regulated and inversely correlated with VEGF expression in CRC [40]. The fact that loss of this region was not correlated with DFS but only with disease recurrence could be due to lack of power for this analysis since loss of this CNA region was observed in 98% of relapse and 74–75% of relapse-free tumors (Figure 1; Supplementary Table S4). Loss of chromosome 4q was previously shown to predict disease relapse in stage II colon cancer patients [13]. This association could not be confirmed in the present series of stage II and III colon cancer samples by the comparison of high-resolution array-CGH profiles from relapse versus non-relapse tumors.

Besides quantitative effects of gene copy numbers related to gained or lost chromosomal segments, also the CNA-associated chromosomal breakpoints may drive oncogenesis by disrupting gene function. Evidence is emerging that genes involved in SVs play an important role in carcinogenesis of epithelial cancers such as CRC [5, 10, 42, 43]. This is one of the first studies that investigated genes that were affected by SVs in a relatively large series of samples using a systematic genome-wide approach. In total, 267 genes that were recurrently affected by CNA-associated breakpoints were identified among 114 MSS colon cancers. Although the resolution of this analysis does not reach nucleotide level, it does illustrate the high prevalence and abundance in which genes may be affected by chromosomal breaks. None of the individual recurrent breakpoint genes were associated with DFS.

In conclusion, differences in prognosis among colon cancer patients must be reflected by inter-tumor heterogeneity regarding somatic DNA alterations, because these aberrations are causal for tumor biology and clinical outcome. The current study showed that mutations in the *APC* tumor suppressor gene that could be detected by TSACP analysis are associated with poor survival of 5-FU-treated stage III MSS colon cancer patients. In addition, synergy of *APC* mutations with activating alterations in MAPK pathway genes was observed. Further validation of the prognostic value of these *APC* mutations in a prospective cohort, as well as mutation analysis of the part of *APC* that was not covered by TSACP analysis in this study, is warranted before these findings could be implemented in a clinical setting.

## MATERIALS AND METHODS

### Sample selection

A total of 57 stage II MSS and 57 stage III MSS colon cancer samples were selected from a single center cohort of 386 patients that underwent radical surgical resection of their primary colon tumor [44]. Tumor selection was performed to allow comparison of non-relapsed *versus* relapsed stage II and III colon cancers, while other clinicopathological parameters were representative for the initial cohort of 386 patients [44]. The stage II cancers comprised 35 non-relapsed and 22 relapsed tumors, and the stage III cancers comprised 30 non-relapsed and 27 relapsed tumors. Staging was done according to the 4th edition of the TNM classification and a relapse was defined by either local or distant tumor recurrence [44]. Median follow-up was 57 months. A detailed overview of clinicopathological characteristics is given in Table 1. Human archival formalin-fixed paraffin-embedded (FFPE) tissue of the primary tumor and matched clinical data was obtained in compliance with the ‘Code for Proper Secondary Use of Human Tissue in The Netherlands’ (www.federa.org).

### Detection of CNAs

DNA was isolated from 10 mm thick FFPE tissue sections as previously described [45]. Tissue sections were macro-dissected to obtain a tumor cell content of at least 70%. Patient-matched normal DNA was isolated from normal mucosa obtained from the resection margin. DNA labeling and hybridization on Agilent 4×180K arrays (Agilent Technologies, Palo Alto, USA) containing 180880 *in situ* synthesized 60-mer oligonucleotides was performed as described previously [46, 47]. This array contained evenly distributed probe locations across the genome with a genomic interval of approximately 17kb, enriched with 4548 additional probes at 238 Cancer Census Gene locations. The array design is available from NCBI's Gene Expression Omnibus (GEO platform GPL8687).

Image acquisition (Microarray scanner G2505B; Agilent technologies) of the arrays and feature extraction (FE software, version 10.5.1.1; Agilent Technologies; protocol CGH_105_Dec08) were carried out with default settings as described by Haan *et al*. [47]. Genomic probe positions on chromosomes 1-22 and X were according to human genome NCBI Build36/hg18. The log2 ratios of tumor versus patient-matched normal pairs were calculated, followed by a wave-smoothing step using “NoWaves” (version 0.4) [48]. Next, log2 ratios were median-normalized and segmented by the Circular Binary Segmentation algorithm [49] (settings: SD-undo: 2 and SD-undo-long: 3) that is incorporated in R-package “DNAcopy” (version 1.38.1), followed by post segmental mode normalization. Segments with posterior probabilities exceeding 0.5 were classified into ‘loss’, ‘gain’ or ‘amplification’ using “CGHcall” (version 2.26.0) [50]. All 114 array-CGH profiles were considered accurate by visual inspection and had median absolute deviation (MAD) values below 0.4. Array-CGH data is deposited in NCBI's Gene Expression Omnibus (GEO accession number GSE75500).

Dimension reduction was achieved by application of “CGHregions” (version 1.22.0; setting: averror: 0.015) [51] and resulted in 808 CNA regions. These regions were subsequently used for two-group comparisons of stage II *versus* III and relapse *versus* non-relapse tumors. Chi-square tests on occurrence of CNA between the two groups were performed including 10000 permutations (CGHtest; www.few.vu.nl/~mavdwiel). Separate tests were performed to compare frequencies of gains *versus* no-gains and losses *versus* no-losses. Associations of CNA regions to DFS were investigated by log-rank test including 10000 permutations using “CGHtest”. To correct for multiple testing, FDR calculation was applied to permutation-based *p*-values. CNA regions observed in less than 10% of samples were excluded from analysis and a FDR less than 0.2 was considered statistically significant [52].

Unsupervised, hierarchical cluster analysis was performed using the R-package “WECCA” (version 0.40) [53] with dendrogram construction based on posterior probabilities [54] (settings: ‘ordinal’, ‘all.equal’, ‘ward’ linkage). For this analysis, the 808 CNA regions were used as input.

### Detection of CNA-associated recurrent breakpoint genes

CNA-associated chromosomal breakpoint detection was performed across the series of 114 array-CGH samples. The 4548 probes used to enrich arrays for probes at Cancer Census Gene locations were excluded for chromosomal breakpoint analysis to obtain an even distribution of probes across the genome [14], leaving a total of 168821 probes (Supplementary Table S1). Consequently, array-CGH data were re-preprocessed using the same settings as for CNA analysis and CNA-associated breakpoints were identified as described previously [14]. The breakpoint detection algorithm has been implemented in an R-package “GeneBreak” (www.bioconductor.org/packages/release/bioc/html/GeneBreak.html) [14]. To assess non-randomness of CNA-associated breakpoint locations statistical analysis was performed with standard Benjamini-Hochberg FDR correction (setting: ‘BH’). CNA-associated chromosomal breakpoint locations were mapped to gene positions that were retrieved by “BiomaRt” (version 2.22.0) and Ensembl (hg18, Ensembl54; Supplementary Table S2) in order to assess what genes were affected by chromosomal breaks. A dedicated Benjamini-Hochberg-type FDR correction [55] was applied (setting: ‘Gilbert’) to identify recurrent breakpoint genes. A FDR less than 0.1 was considered significant [14].

### Detection of gene mutations

Mutation status of *APC*, *TP53*, *KRAS*, *PIK3CA*, *FBXW7*, *SMAD4*, *BRAF* and *NRAS*, *i.e.* genes that are commonly mutated in CRC, was assessed by next generation sequencing analysis of FFPE DNA samples using the TruSeq Amplicon Cancer Panel (TSACP; Illumina Inc, San Diego, CA USA). Reads were aligned to the human reference genome (NCBI Build37/hg19). “Falco” [56] was used to detect DNA aberrations that were called as a mutation when observed in at least 20% of the reads, not classified as synonymous mutations and not present in dbSNP (build 137). A total of 60 samples reached quality criteria for gene mutation analysis [56], whereby the distribution of main clinical characteristics of these 60 samples were comparable to the series of 114 samples (Supplementary Table S3). Visualization of mutation events across samples was generated using “OncoPrinter” [57, 58].

### Determination of a CNA- and BP-score

The CNAs were quantified and expressed as a ‘CNA-score’, which represents the number of array-CGH probes that deviated from being copy number neutral. In addition, the ‘BP-score’ was used to represent the number of CNA-associated chromosomal breakpoint locations. [59] CNA-scores and BP-scores of subgroups of cancer samples that were stratified for *TP53* and *APC* mutation status were compared using two-sided Mann-Whitney *U* tests.

### Survival analysis

Associations of gene mutation status with disease-free survival (DFS) were evaluated by univariate Kaplan-Meier analysis. Survival rates were visualized by Kaplan-Meier curves and compared using a two-sided log-rank test (univariate). Hazard ratios (HR) were calculated using Cox regression analysis.

## SUPPLEMENTARY MATERIALS FIGURES AND TABLES










