# Supplementary material for: Genomic profiling of stage II and III colon cancers reveals APC mutations to be associated with survival in stage III colon cancer patients
Source: Oncotarget. 2016 Oct 6;7(45):73876–87. doi: 10.18632/oncotarget.12510 (PMC5342020; doi:10.18632/oncotarget.12510)
Supplement: Supplementary file 1 [file oncotarget-07-73876-s001.pdf]

Genomic profiling of stage II and III colon cancers reveals *APC* mutations to be associated with survival in stage III colon cancer patients

Supplementary Materials

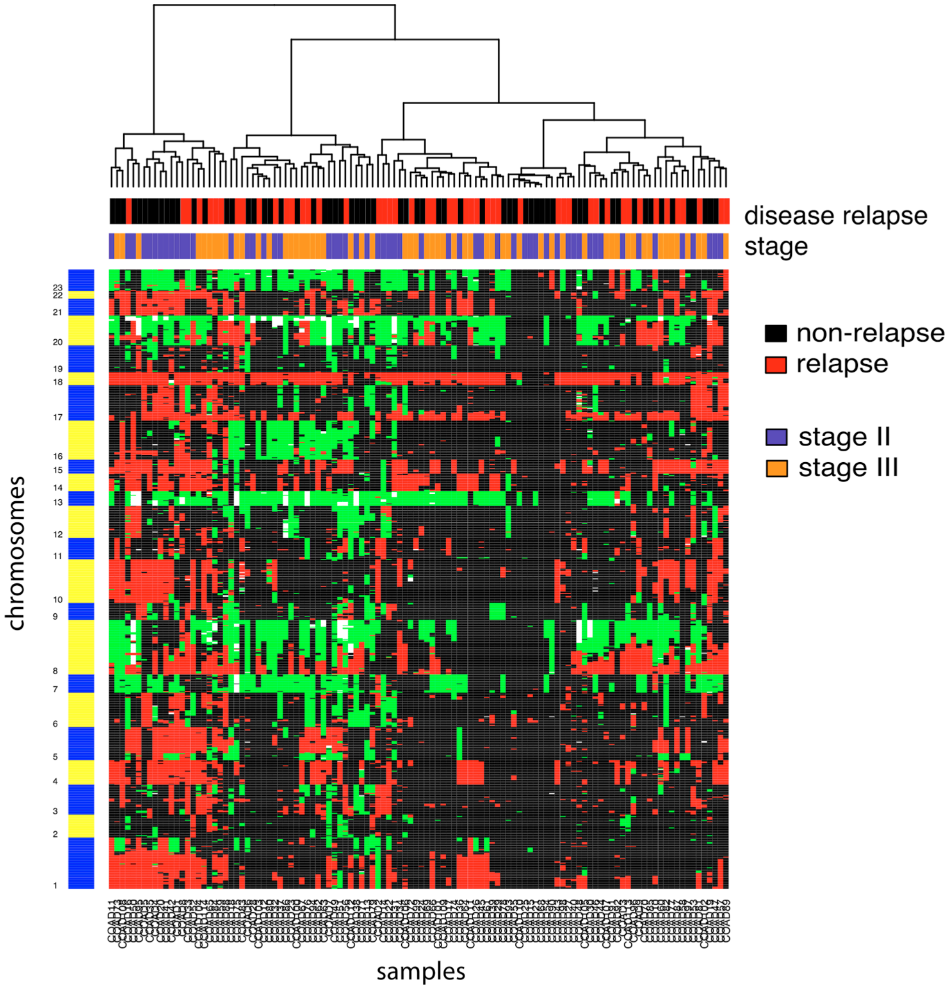

Supplementary Figure S1: Dendrogram of unsupervised hierarchical clustering and heatmap of 808 CNA regions from stage II and III colon cancer samples (*n* = 114). The samples are ordered on the horizontal axis and the vertical axis reflects chromosomal location ordered from 1-22 and X (numbered 23). Odd chromosomes are depicted in blue and even chromosomes in yellow. Within the heatmap, black depict normal copy number regions, red regions losses, green regions gains and white regions amplifications. Clinicopathological information with respect to disease recurrence and tumor stage are indicated by two horizontal bars. Tumors that did or did not develop a relapse are depicted in red and black respectively. Stage II colon cancer patients are depicted in purple and stage III in orange.

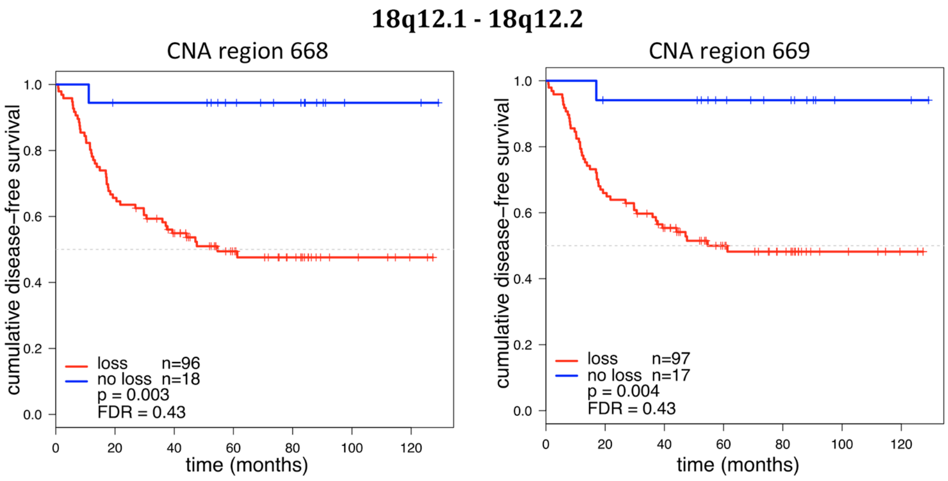

Supplementary Figure S2: Kaplan-Meier curves for disease-free survival (in months) of stage II and stage III colon cancers (n = 114) stratified for copy number 'loss' versus 'no loss' of chromosome 18q12.1-18q12.2 (CNA regions 668-669; Supplementary Table S4). *P*-values and FDR's were obtained by permutation-based analysis ("CGHtest"; Supplementary Table S4). There was no significant association between these CNA regions and DFS (threshold for significance set to FDR of 0.2 [1]).

REFERENCE

1. Voorham QJM, Carvalho B, Spiertz AJ, van Grieken NCT, Mongera S, Rondagh EJA, van de Wiel MA, Jordanova ES, Ylstra B, Kliment M, Grabsch H, Rembacken BJ, Arai T, et al. Chromosome 5q loss in colorectal flat adenomas. Clin Cancer Res. 2012; 18:4560–9.

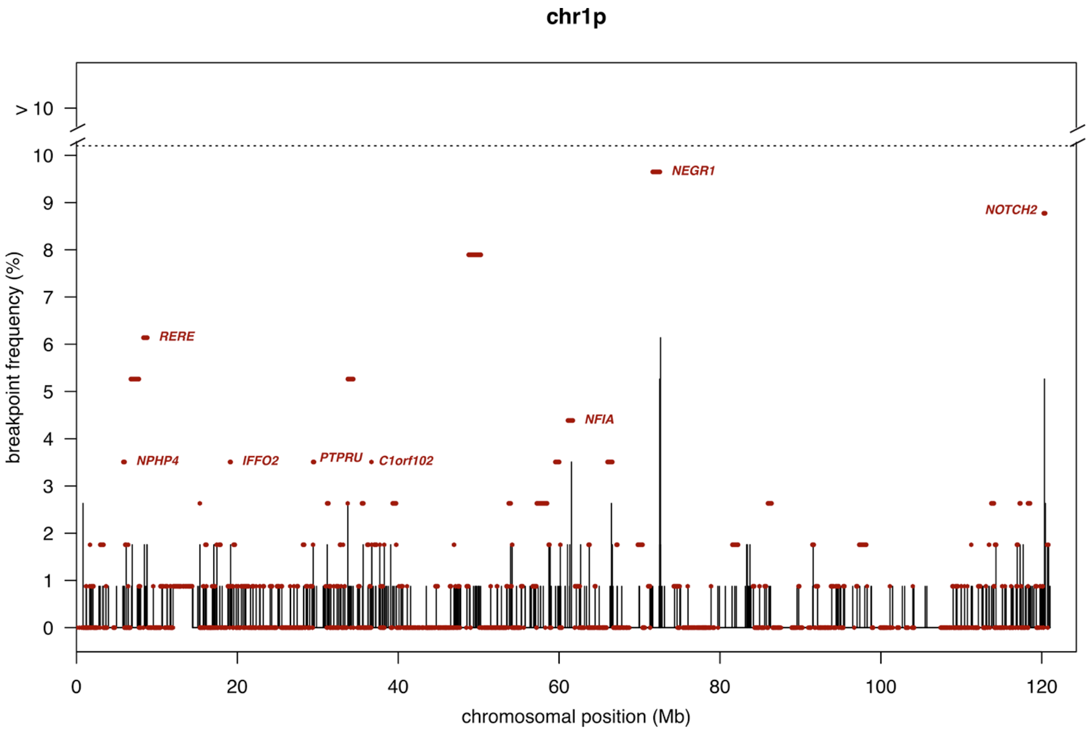

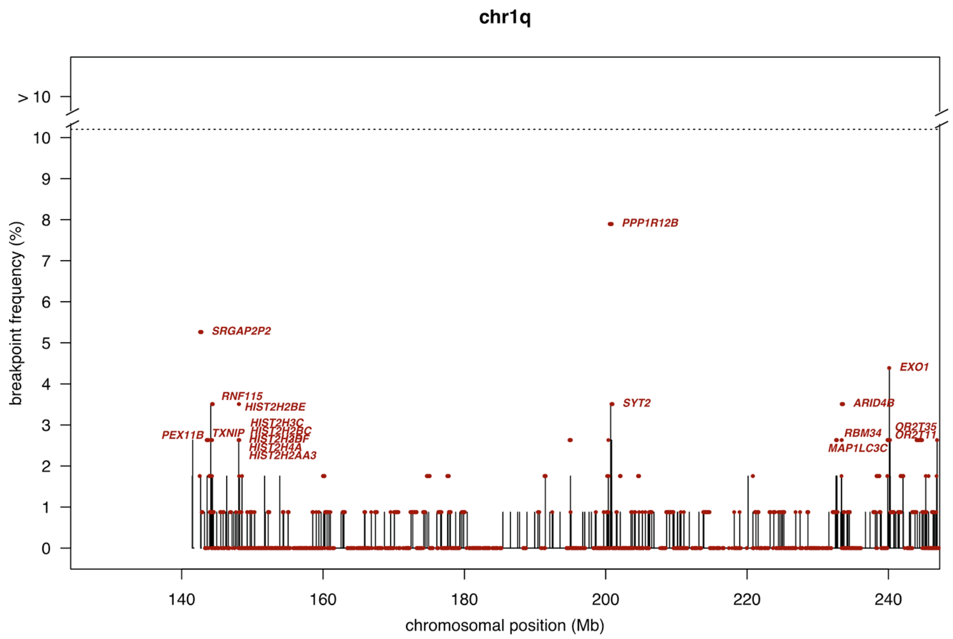
chr1q
> 10
10
9
8
7
6
5
4
3
2
1
0
breakpoint frequency (%)
PPP1R12B
SRGAP2P2
EXO1
RNF115
HIST2H2BE
SYT2
ARID4B
HIST2H3C
HIST2H2BC
HIST2H3BF
HIST2H4A
HIST2H2AA3
PEX11B
TXNIP
RBM34
MAP1LC3C
OR2T35
OR2T11
140
160
180
200
220
240
chromosomal position (Mb)

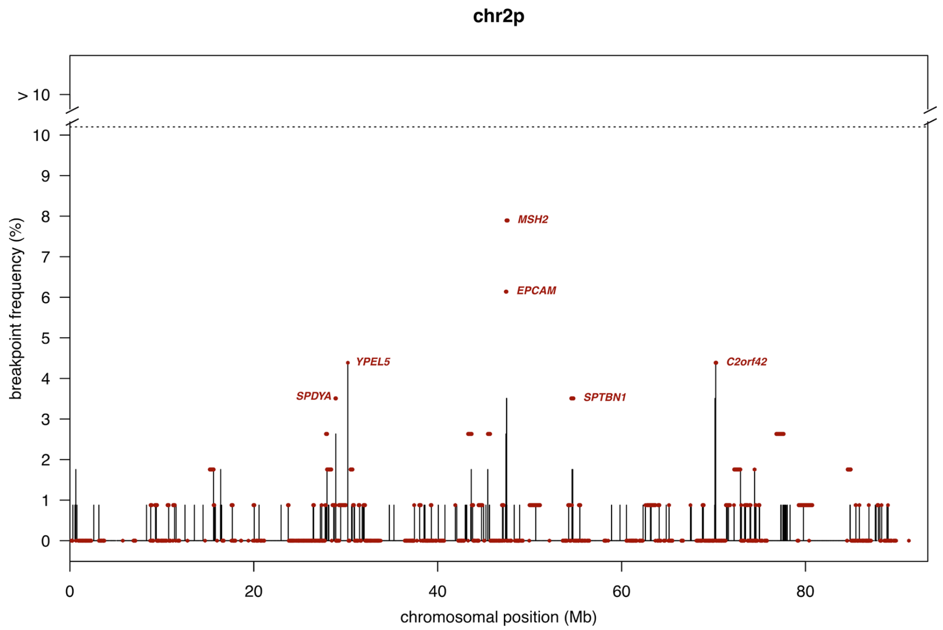
chr2p
> 10
10
9
8
7
6
5
4
3
2
1
0
breakpoint frequency (%)
MSH2
EPCAM
YPEL5
C2orf42
SPDYA
SPTBN1
0
20
40
60
80
chromosomal position (Mb)

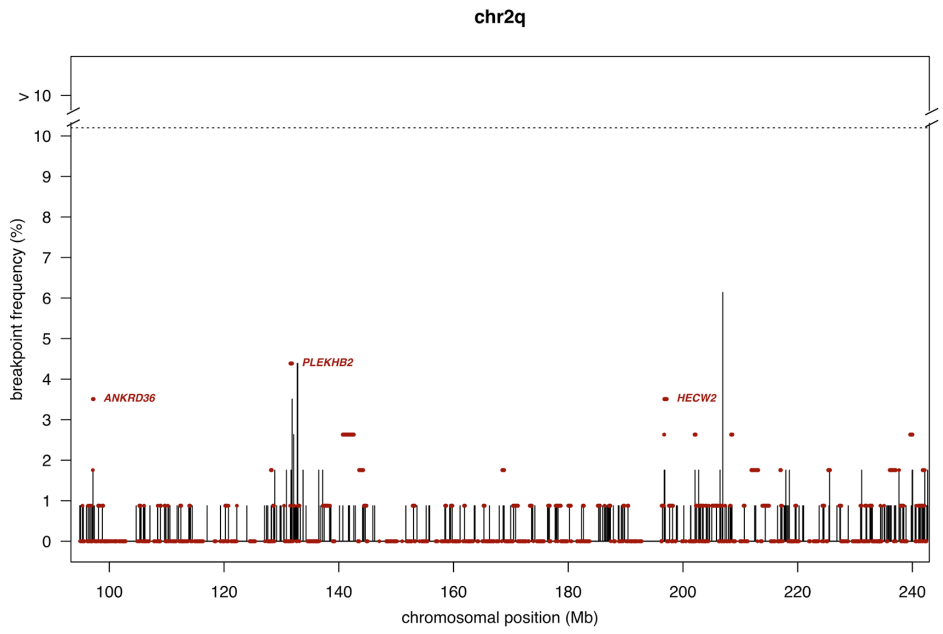
chr2q
> 10
10
9
8
7
6
5
4
3
2
1
0
breakpoint frequency (%)
PLEKHB2
ANKRD36
HECW2
100
120
140
160
180
200
220
240
chromosomal position (Mb)

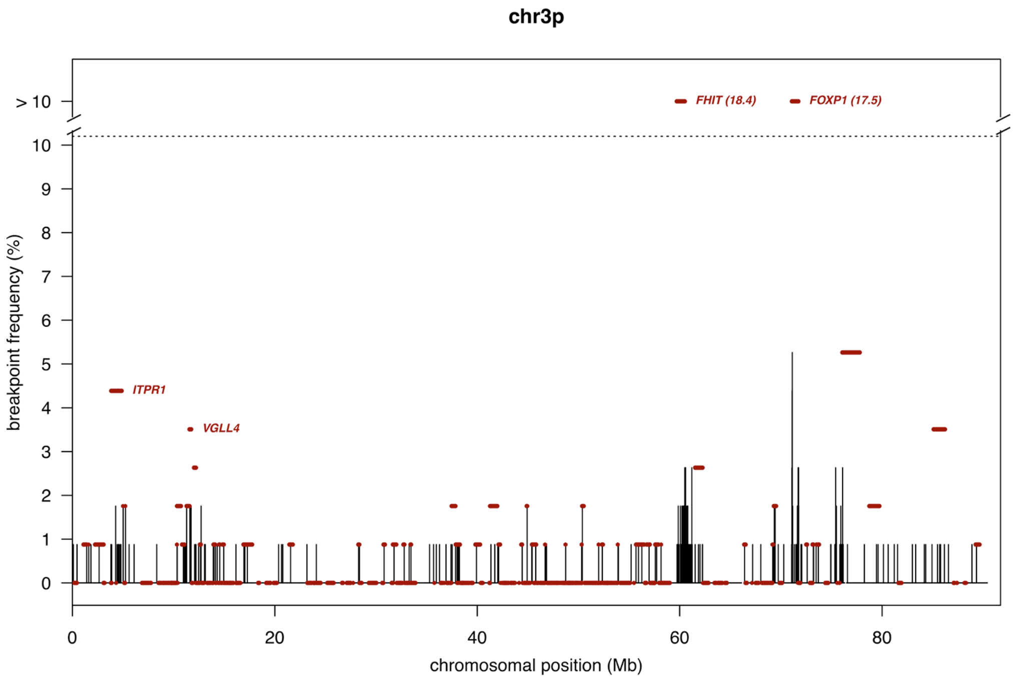
chr3p
> 10
FHIT (18.4)
FOXP1 (17.5)
breakpoint frequency (%)
ITPR1
VGLL4
0
1
2
3
4
5
6
7
8
9
10
0
20
40
60
80
chromosomal position (Mb)

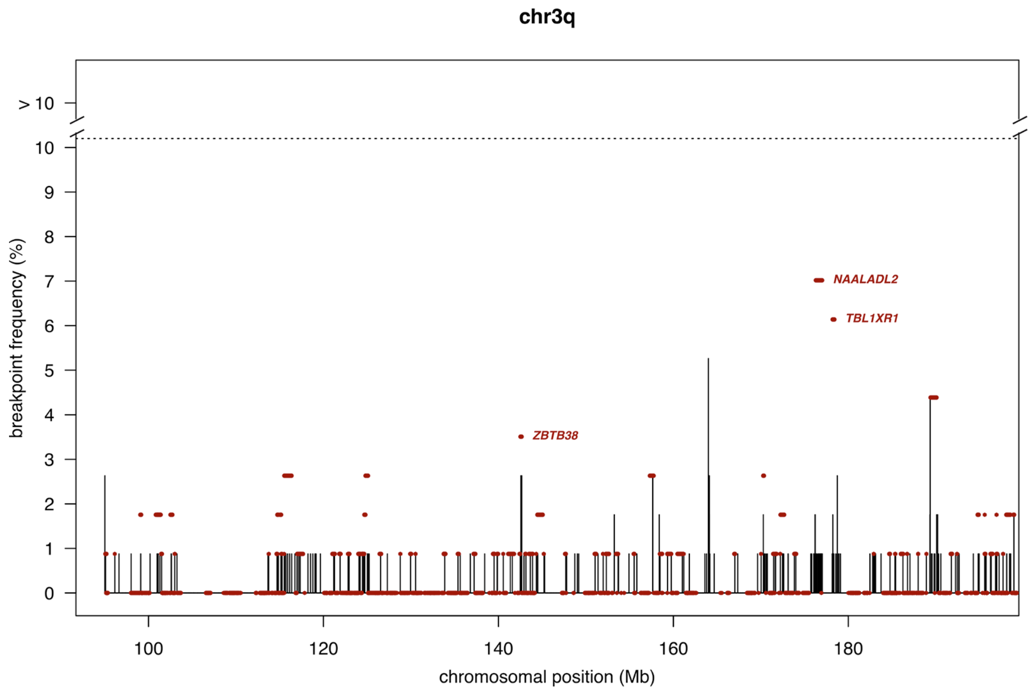
chr3q
> 10
breakpoint frequency (%)
NAALADL2
TBL1XR1
ZBTB38
0
1
2
3
4
5
6
7
8
9
10
100
120
140
160
180
chromosomal position (Mb)

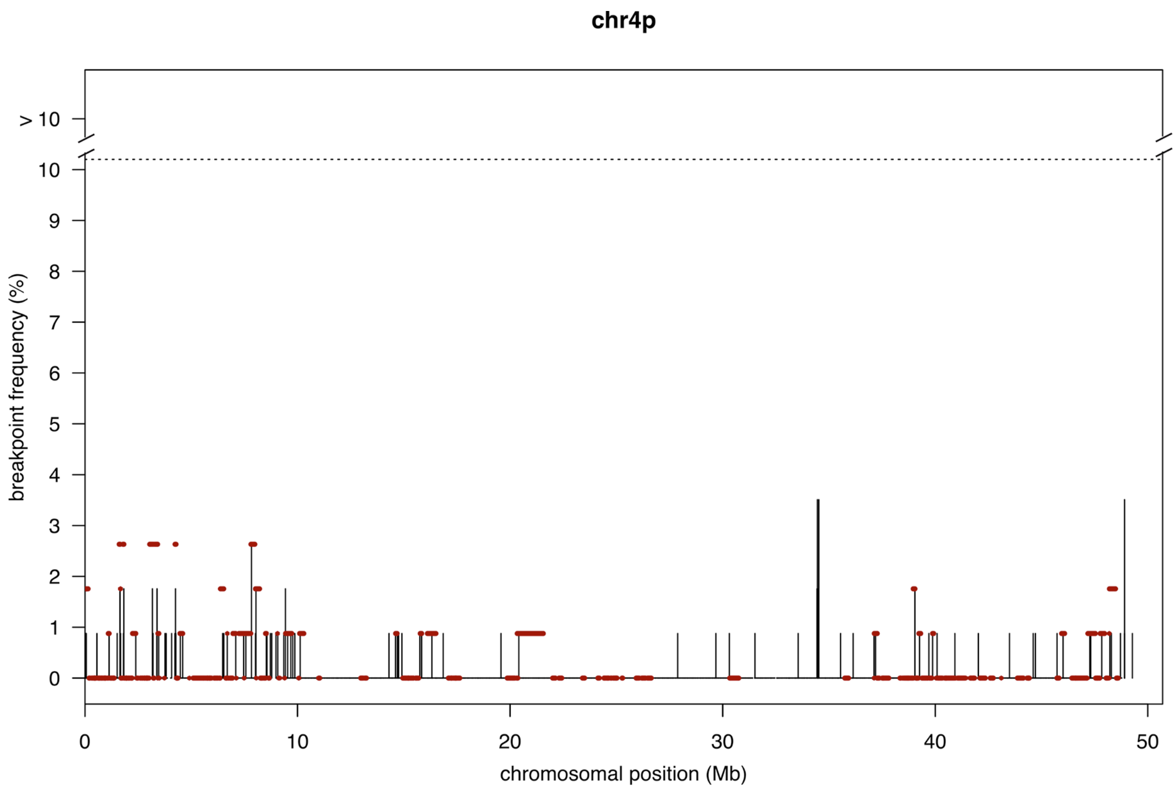
chr4p
> 10
10
9
8
7
6
5
4
3
2
1
0
breakpoint frequency (%)
0
10
20
30
40
50
chromosomal position (Mb)

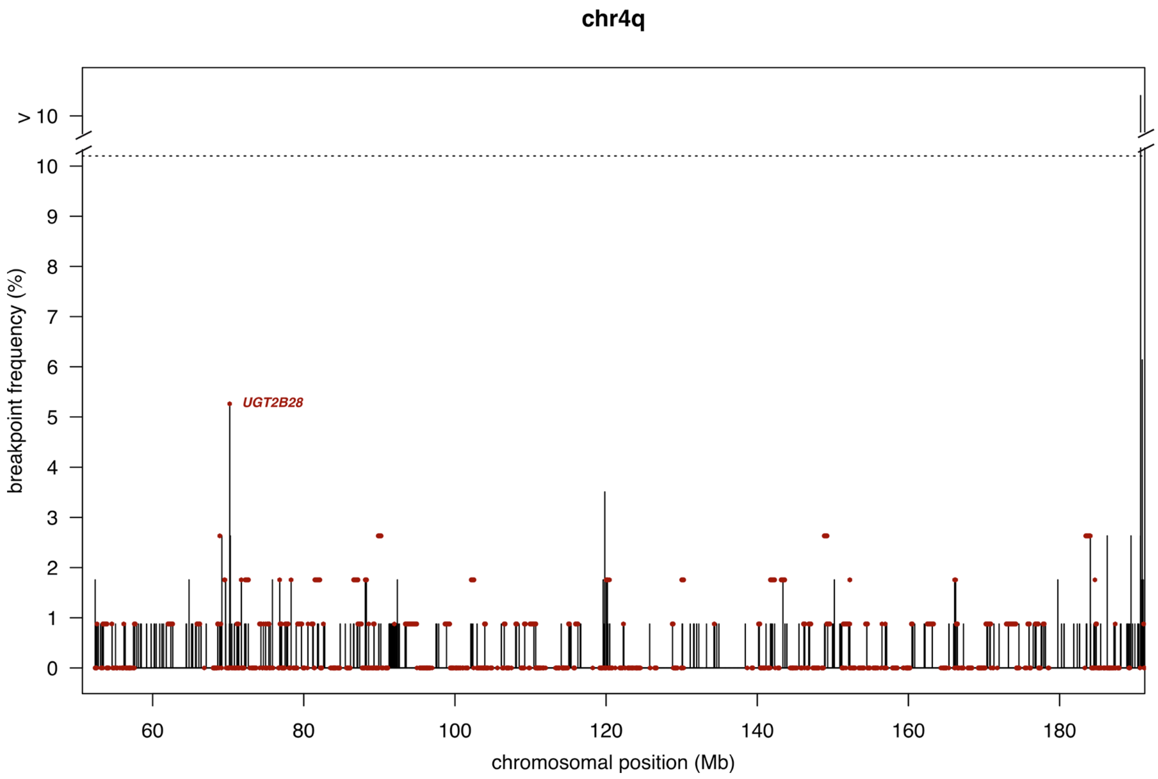
chr4q
> 10
10
9
8
7
6
5
4
3
2
1
0
breakpoint frequency (%)
UGT2B28
60
80
100
120
140
160
180
chromosomal position (Mb)

chr5p

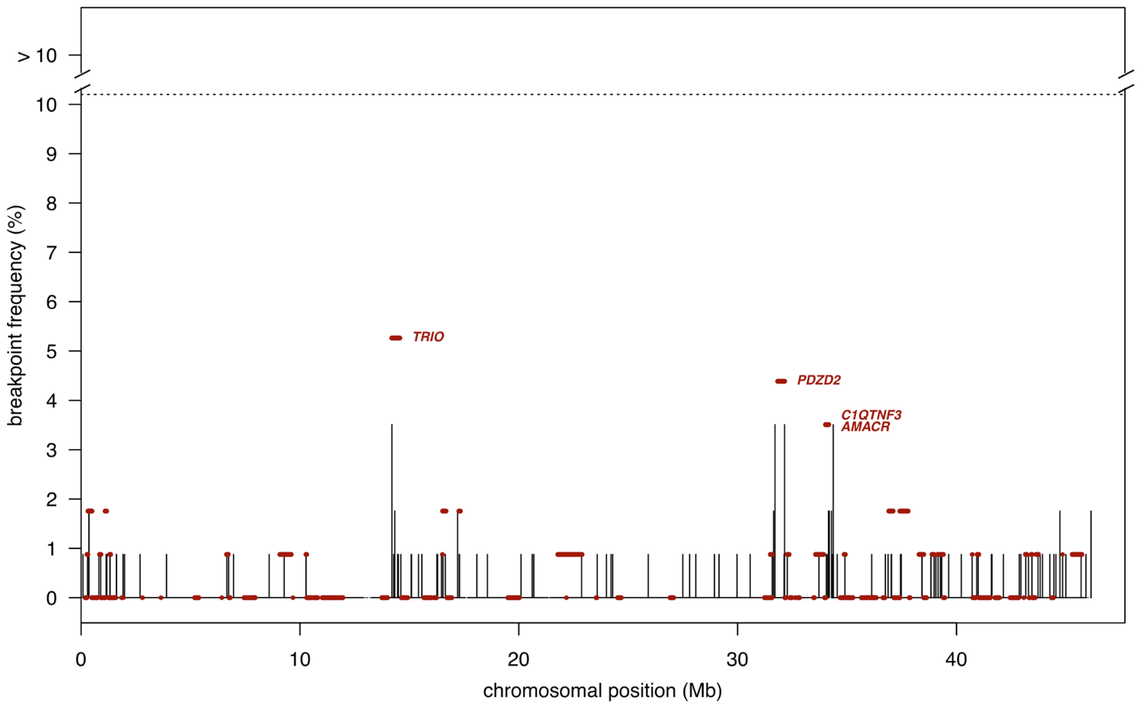

> 10
breakpoint frequency (%)
chromosomal position (Mb)
TRIO
PDZD2
C1QTNF3
AMACR

chr5q

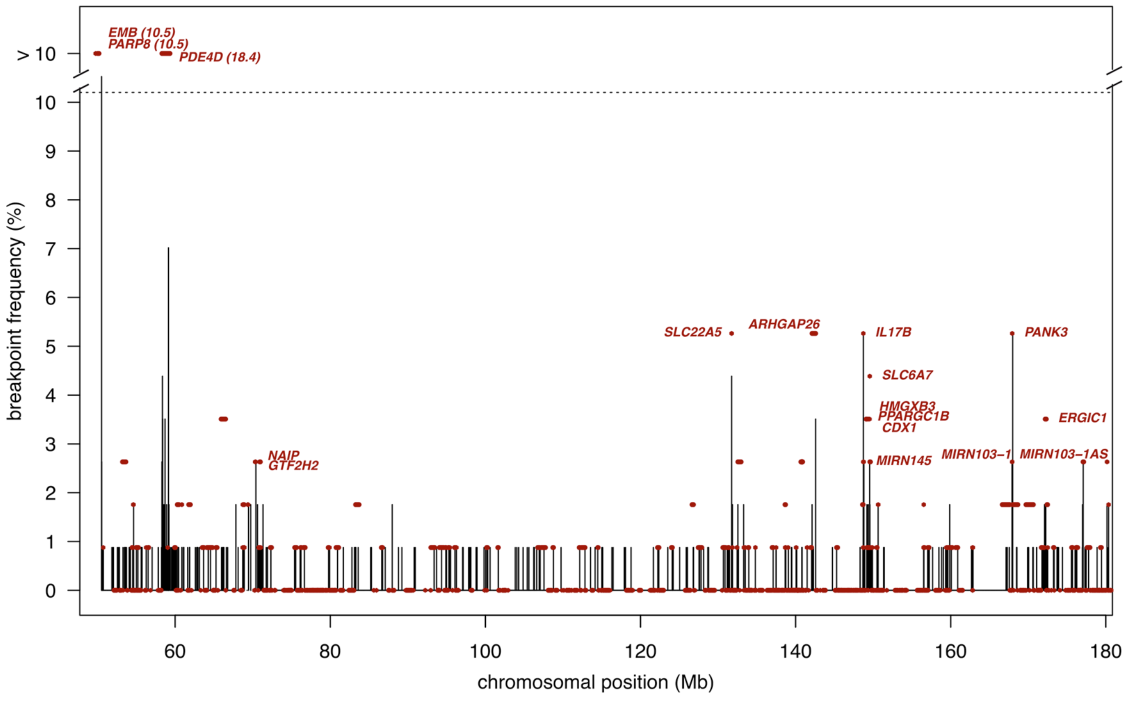

> 10
EMB (10.5)
PARP8 (10.5)
PDE4D (18.4)
breakpoint frequency (%)
chromosomal position (Mb)
NAIP
GTF2H2
SLC22A5
ARHGAP26
IL17B
SLC6A7
HMGXB3
PPARGC1B
CDX1
MIRN145
MIRN103−1
MIRN103−1AS
PANK3
ERGIC1

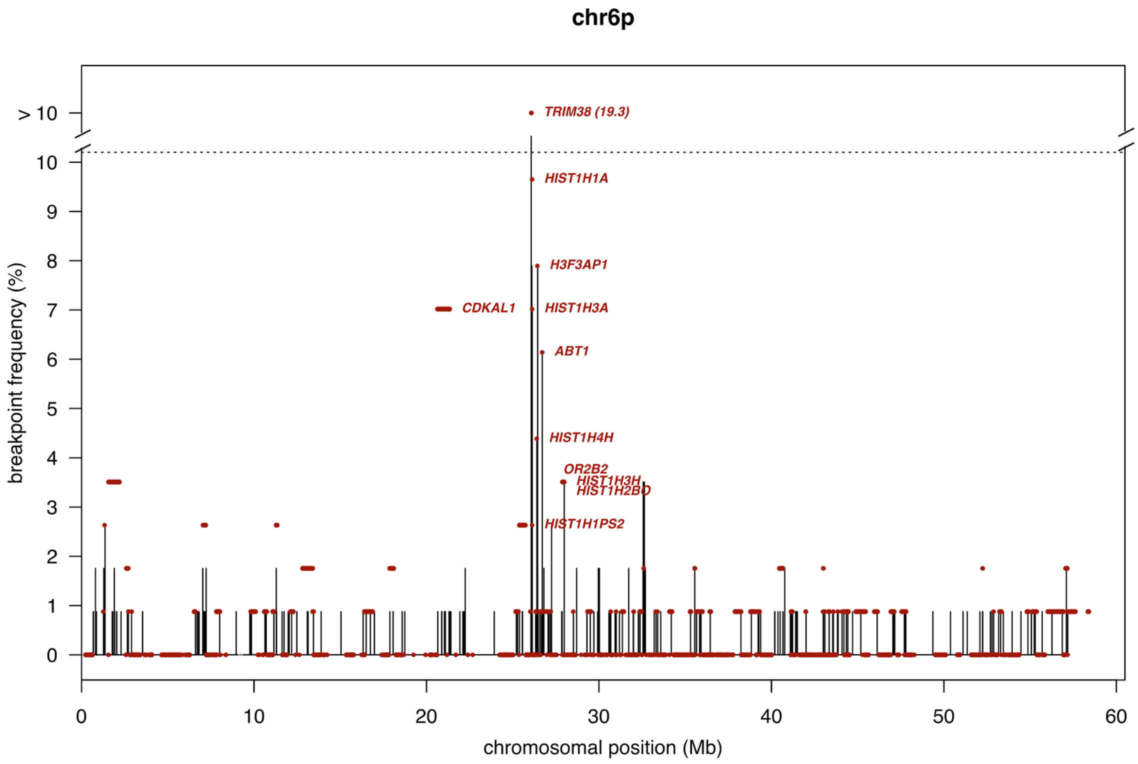
chr6p
> 10
TRIM38 (19.3)
HIST1H1A
H3F3AP1
CDKAL1
HIST1H3A
ABT1
HIST1H4H
OR2B2
HIST1H3H
HIST1H2BO
HIST1H1PS2
breakpoint frequency (%)
chromosomal position (Mb)
0 1 2 3 4 5 6 7 8 9 10
0 10 20 30 40 50 60

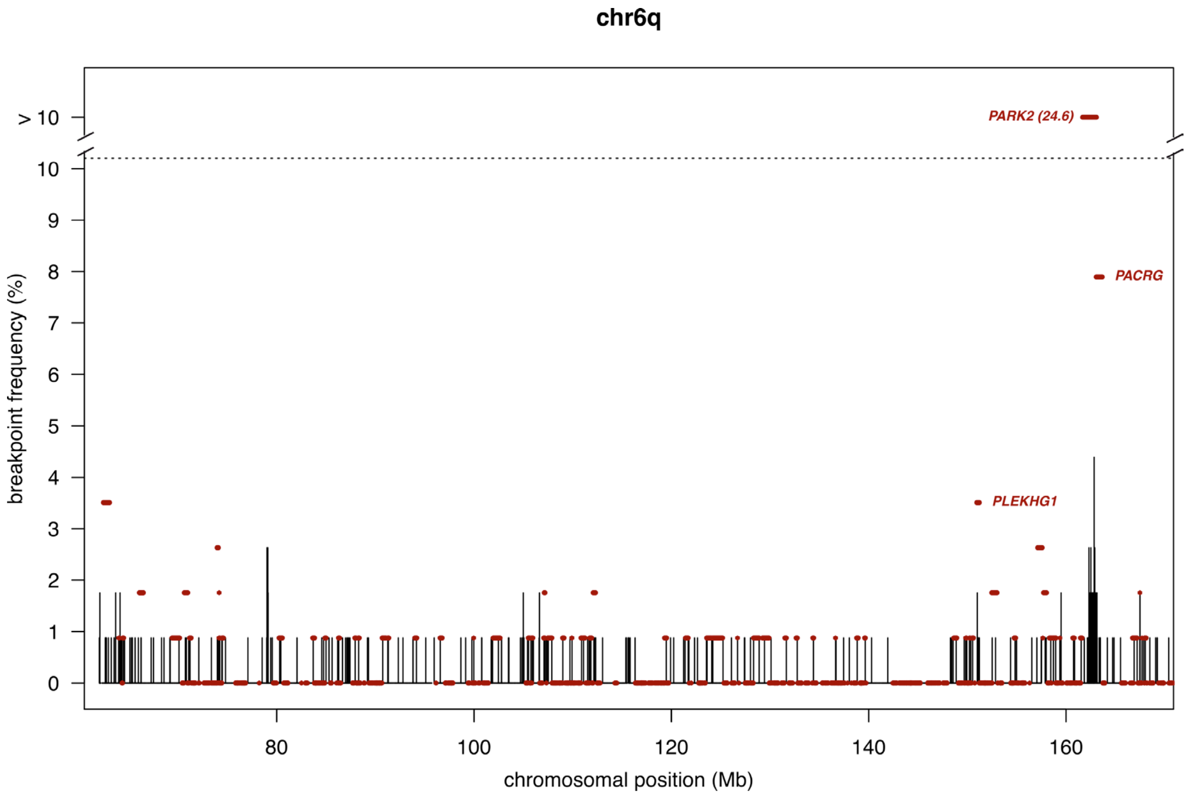
chr6q
> 10
PARK2 (24.6)
PACRG
PLEKHG1
breakpoint frequency (%)
chromosomal position (Mb)
0 1 2 3 4 5 6 7 8 9 10
80 100 120 140 160

chr7p

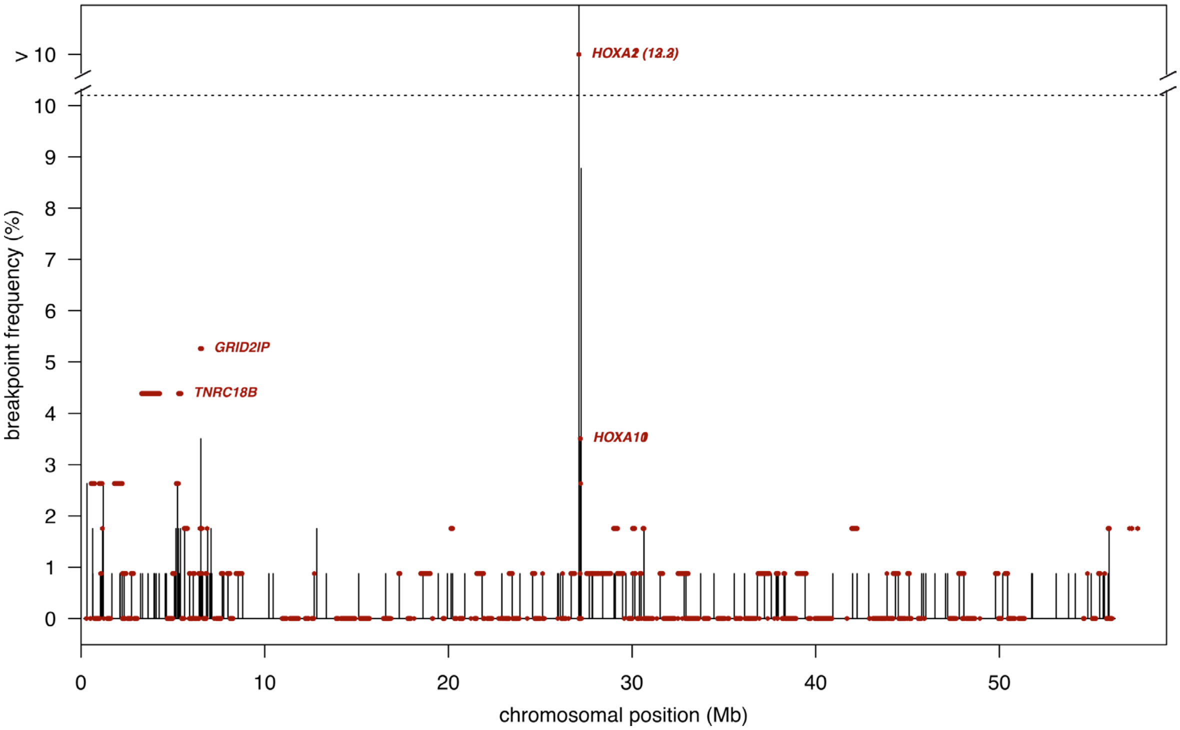

HOXA2 (13.3)
GRID2IP
TNRC18B
HOXA10
> 10
breakpoint frequency (%)
chromosomal position (Mb)

chr7q

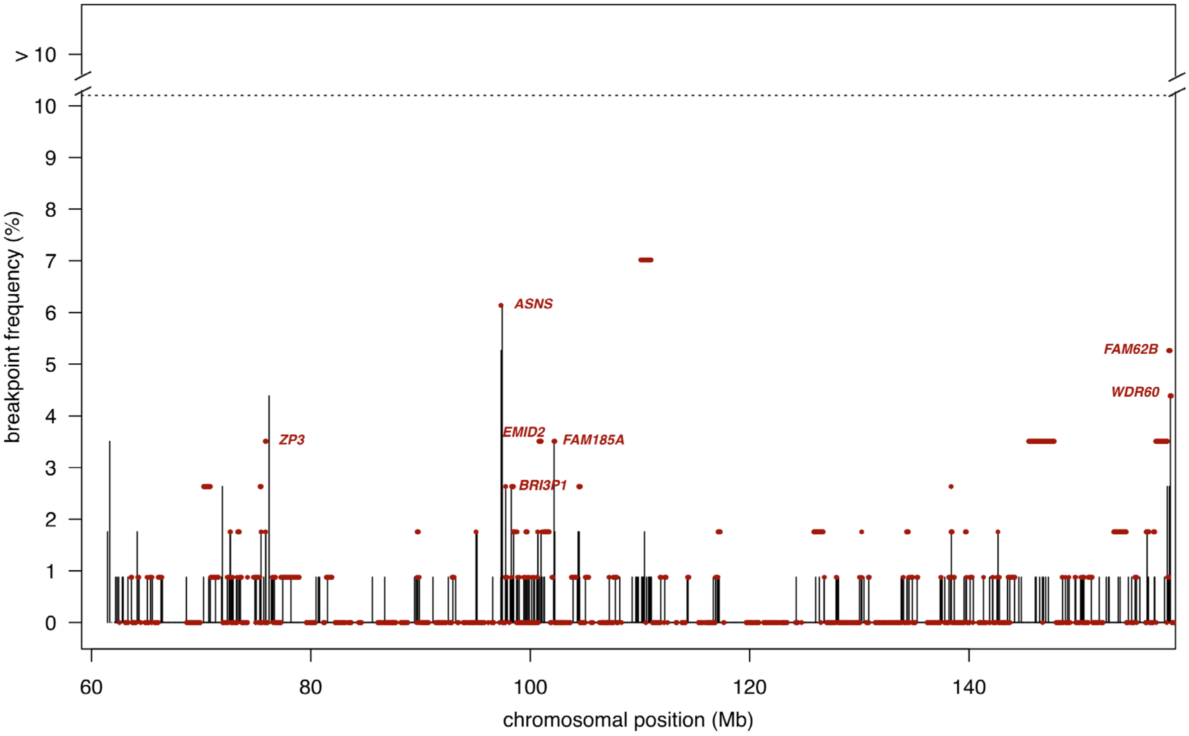

ASNS
FAM62B
WDR60
ZP3
EMID2
FAM185A
BRI3P1
> 10
breakpoint frequency (%)
chromosomal position (Mb)

chr8p

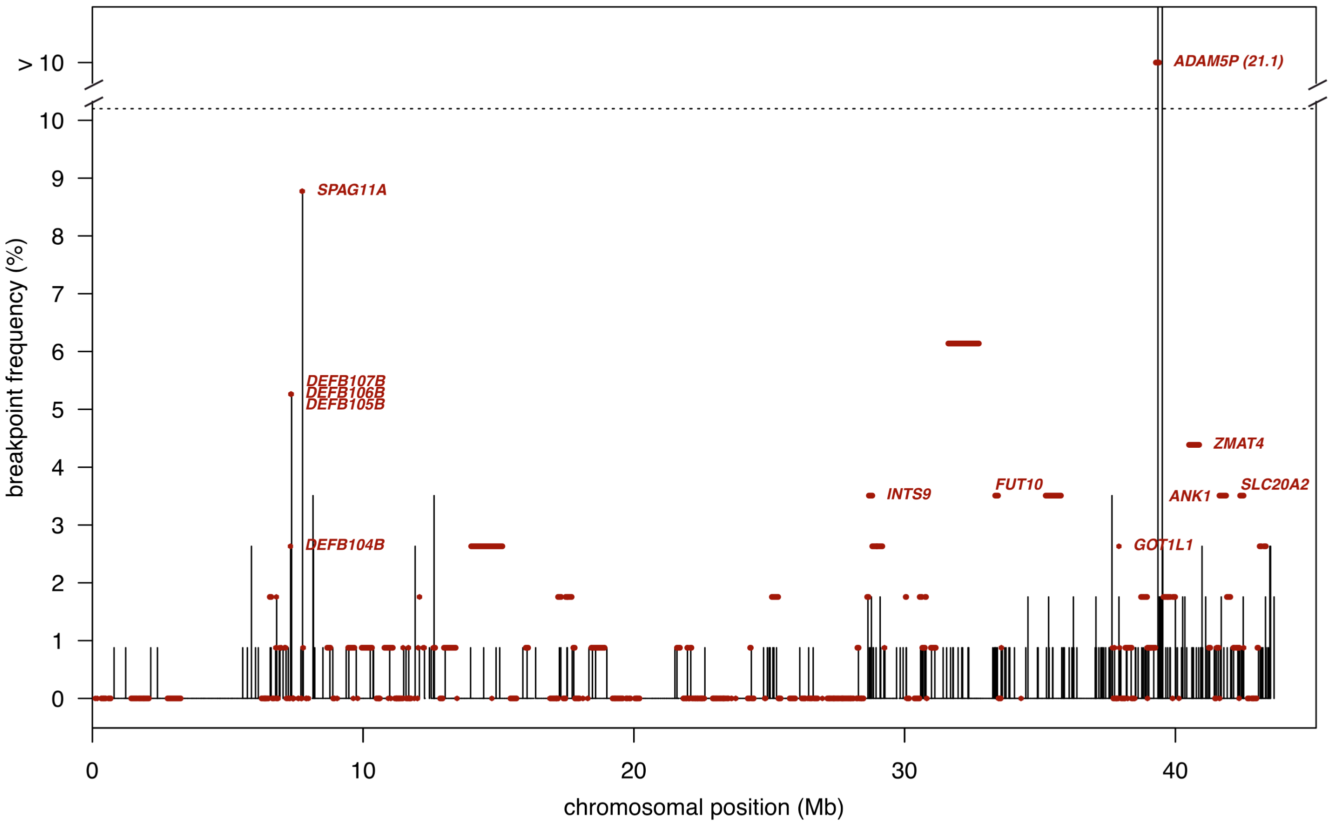
ADAM5P (21.1)
SPAG11A
DEFB107B
DEFB106B
DEFB105B
DEFB104B
INTS9
FUT10
ZMAT4
ANK1
SLC20A2
GOT1L1
breakpoint frequency (%)
chromosomal position (Mb)

chr8q

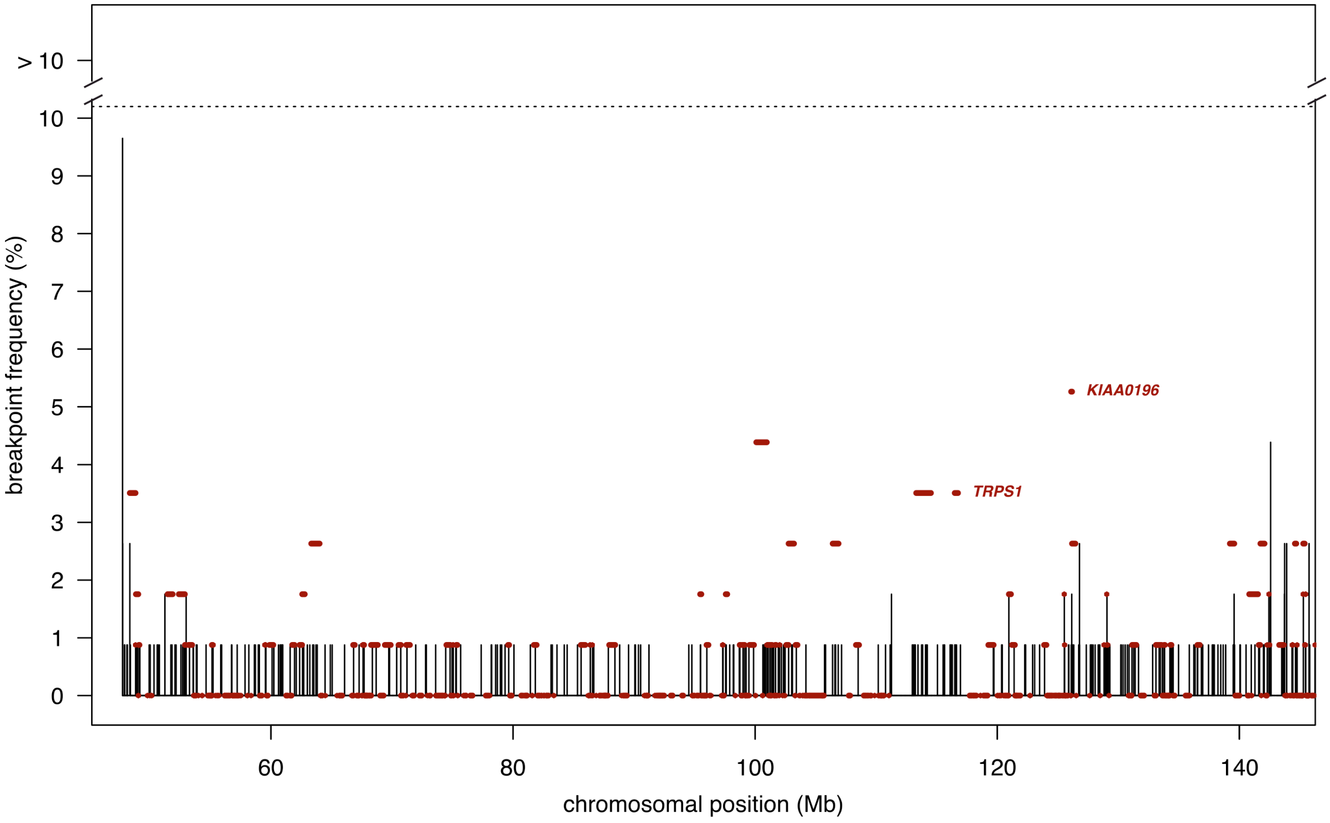
KIAA0196
TRPS1
breakpoint frequency (%)
chromosomal position (Mb)

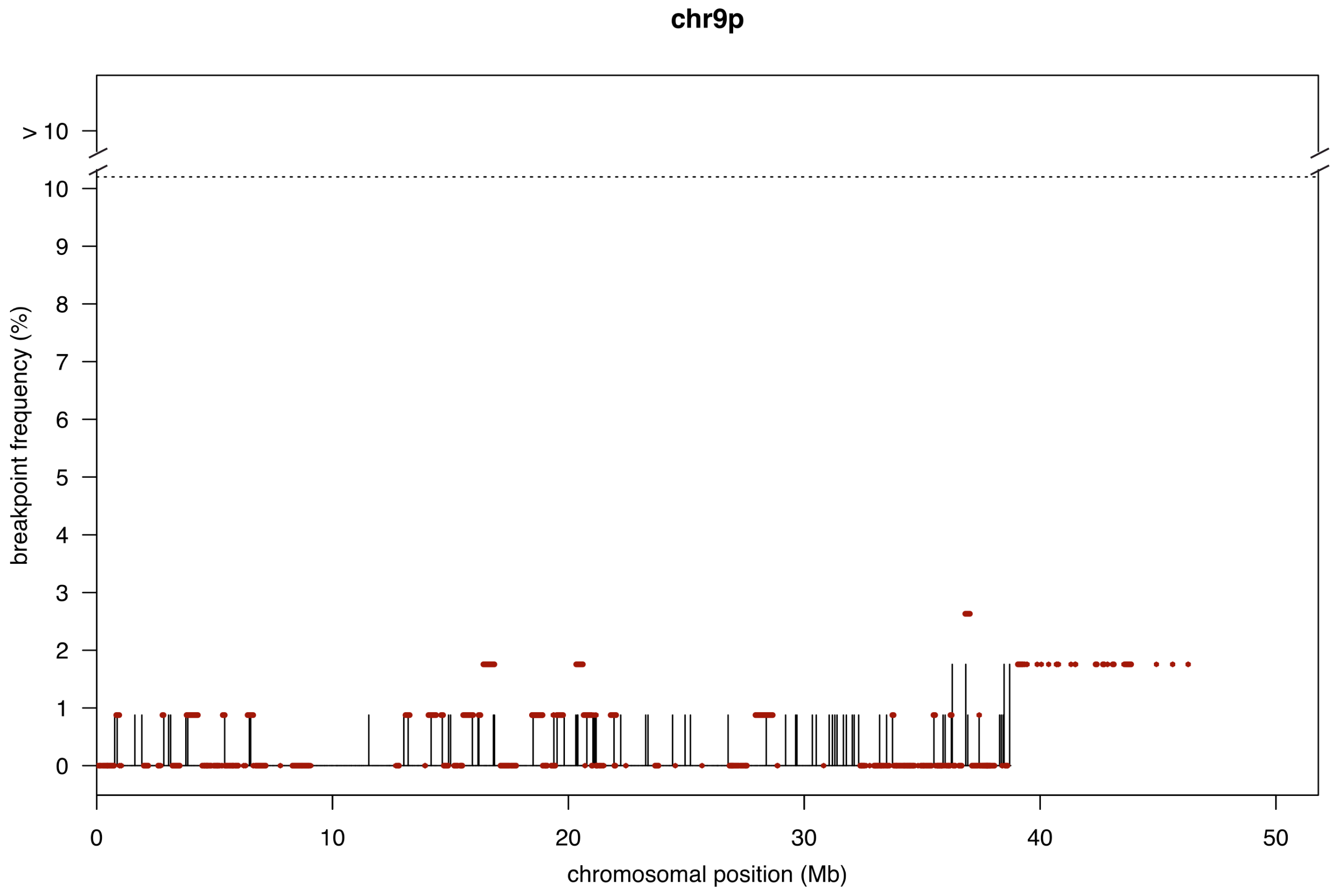
chr9p
> 10
10
9
8
7
6
5
4
3
2
1
0
breakpoint frequency (%)
0
10
20
30
40
50
chromosomal position (Mb)

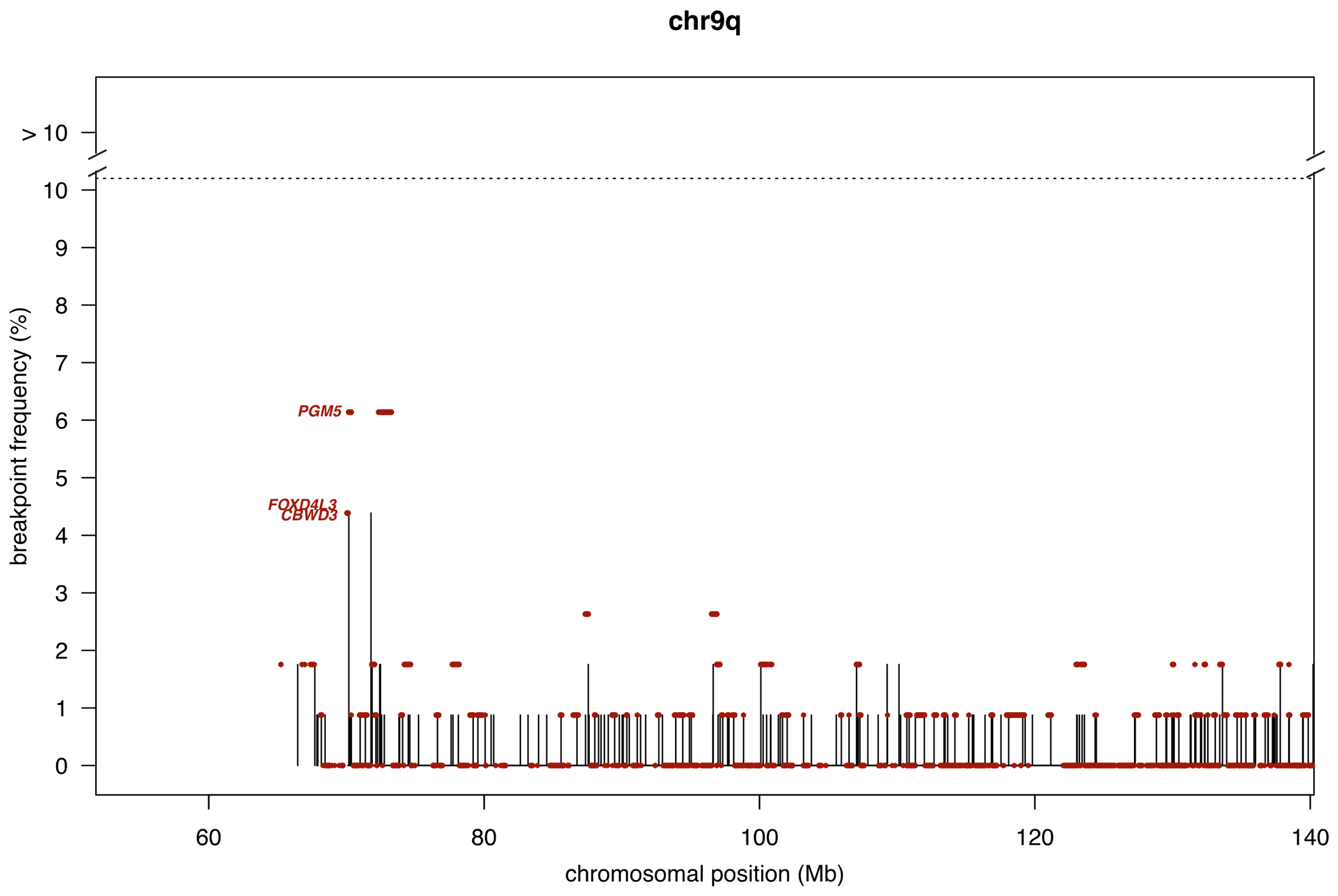
chr9q
> 10
10
9
8
7
6
5
4
3
2
1
0
breakpoint frequency (%)
PGM5
FOXD4L3
CBWD3
60
80
100
120
140
chromosomal position (Mb)

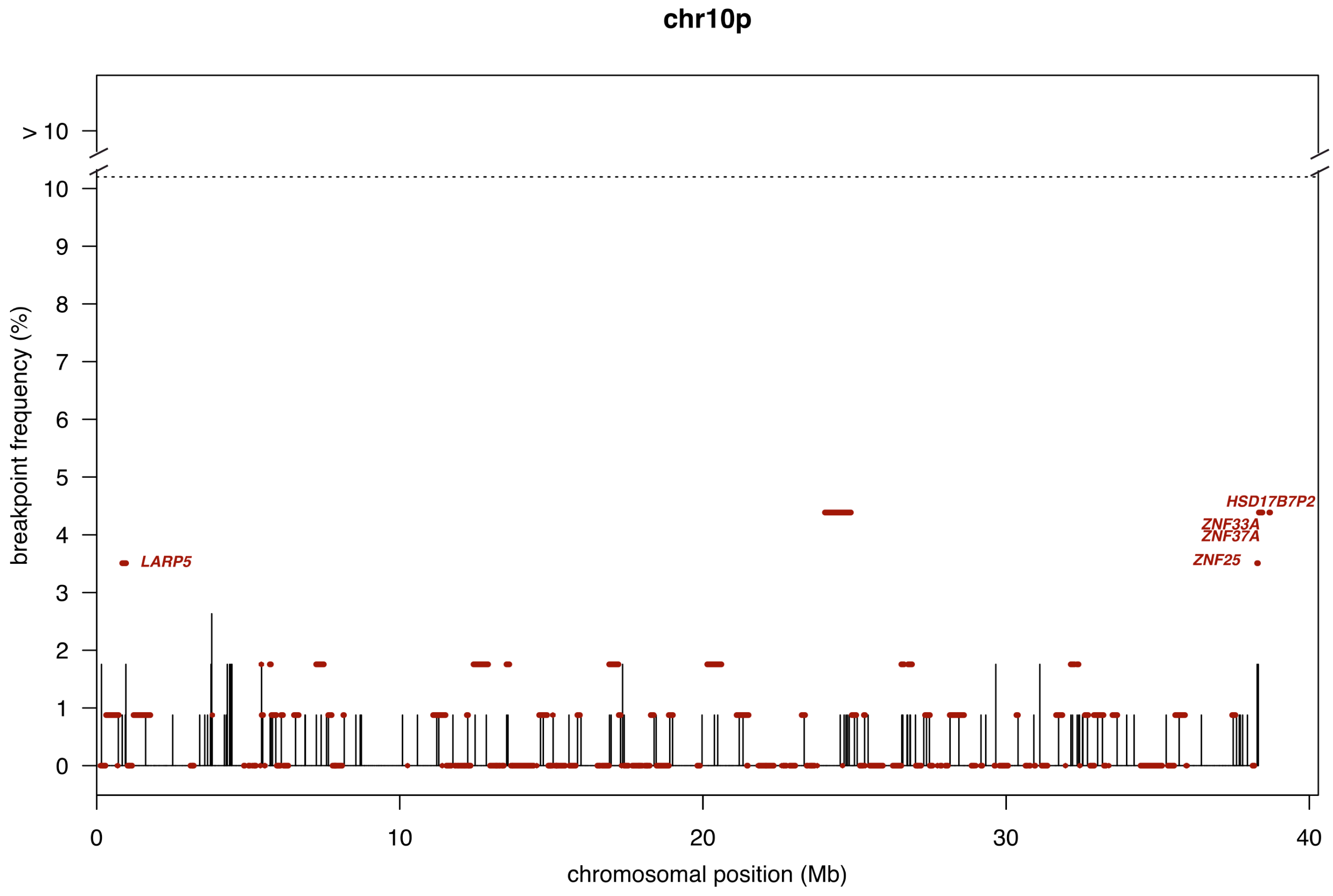
chr10p
> 10
10
9
8
7
6
5
4
3
2
1
0
breakpoint frequency (%)
LARP5
HSD17B7P2
ZNF33A
ZNF37A
ZNF25
0
10
20
30
40
chromosomal position (Mb)

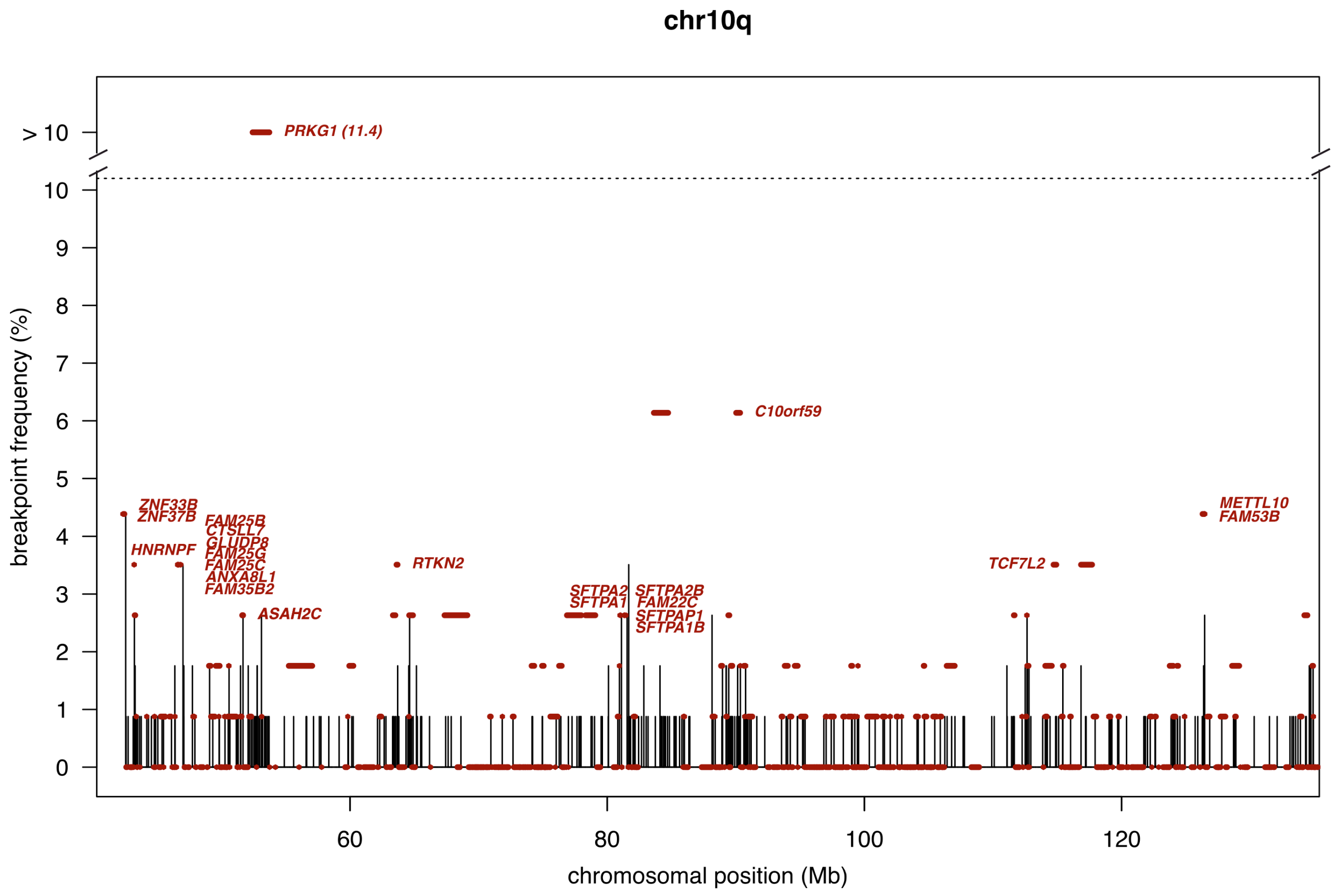
chr10q
> 10
PRKG1 (11.4)
10
9
8
7
6
5
4
3
2
1
0
breakpoint frequency (%)
C10orf59
ZNF33B
ZNF37B
FAM25B
CTSLL7
GLUDP8
HNRNPF
FAM25G
FAM25C
ANXA8L1
FAM35B2
ASAH2C
RTKN2
SFTPA2
SFTPA1
SFTPA2B
FAM22C
SFTPAP1
SFTPA1B
TCF7L2
METTL10
FAM53B
60
80
100
120
chromosomal position (Mb)

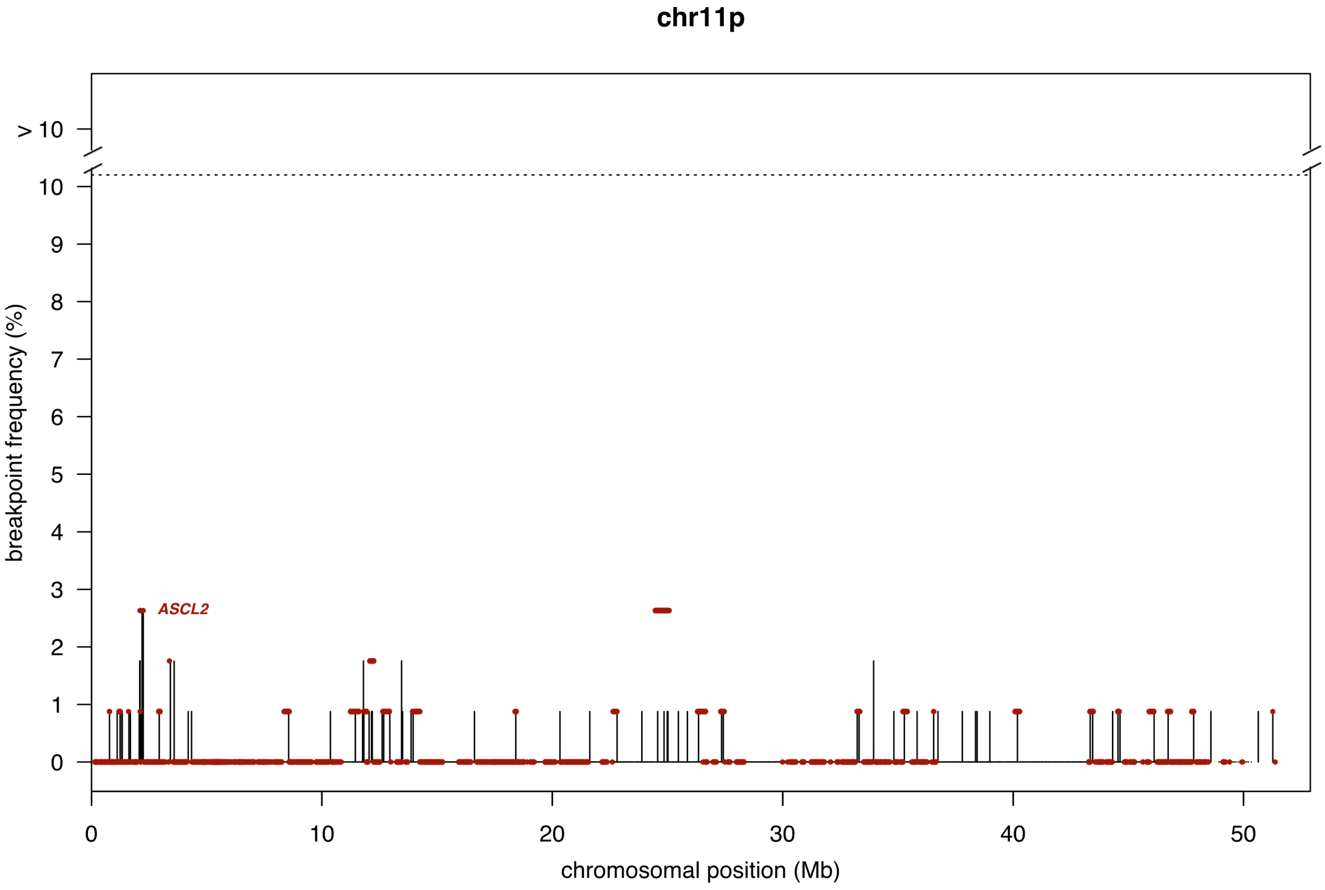
chr11p
> 10
breakpoint frequency (%)
ASCL2
chromosomal position (Mb)

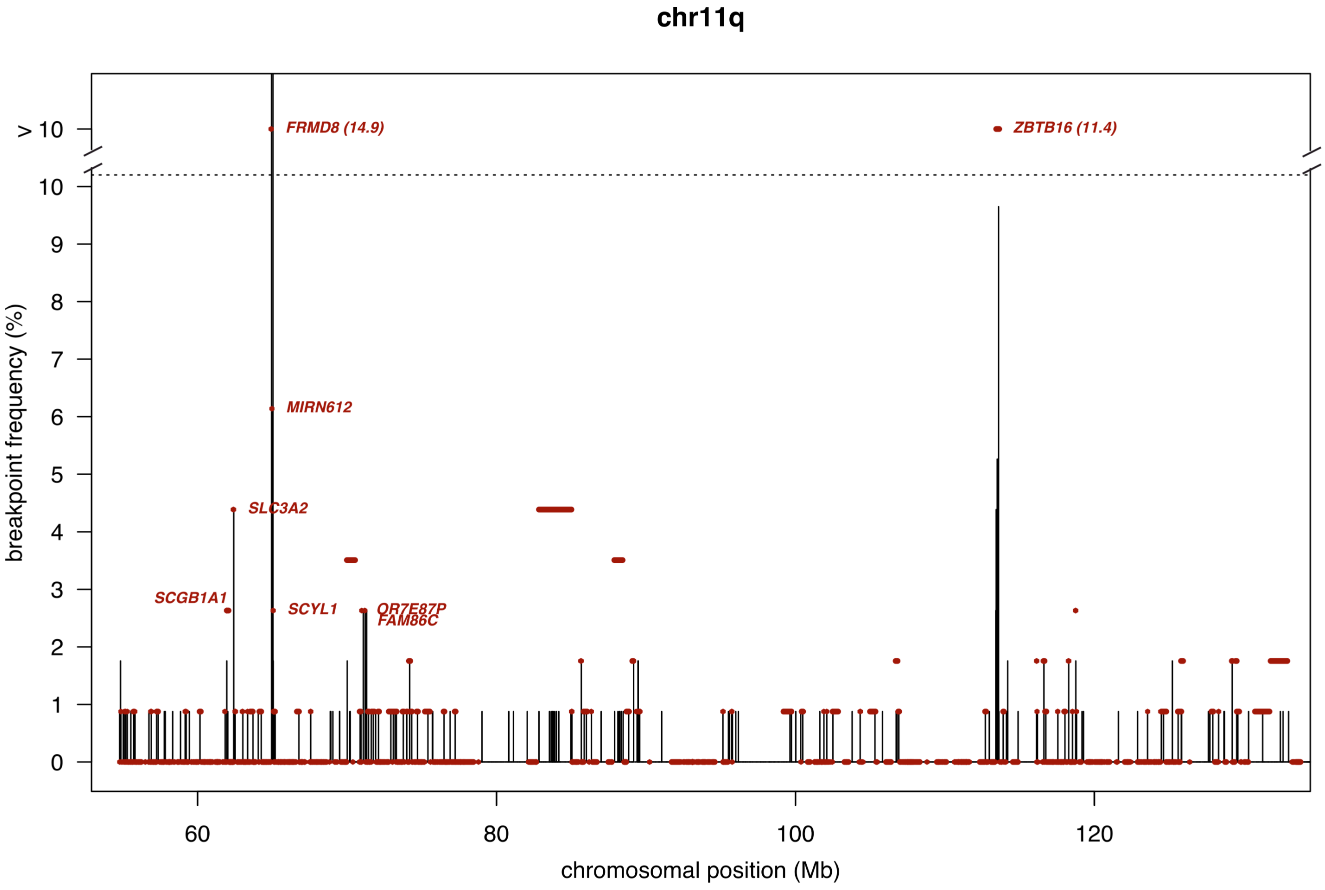
chr11q
> 10
FRMD8 (14.9)
ZBTB16 (11.4)
breakpoint frequency (%)
MIRN612
SLC3A2
SCGB1A1
SCYL1
OR7E87P
FAM86C
chromosomal position (Mb)

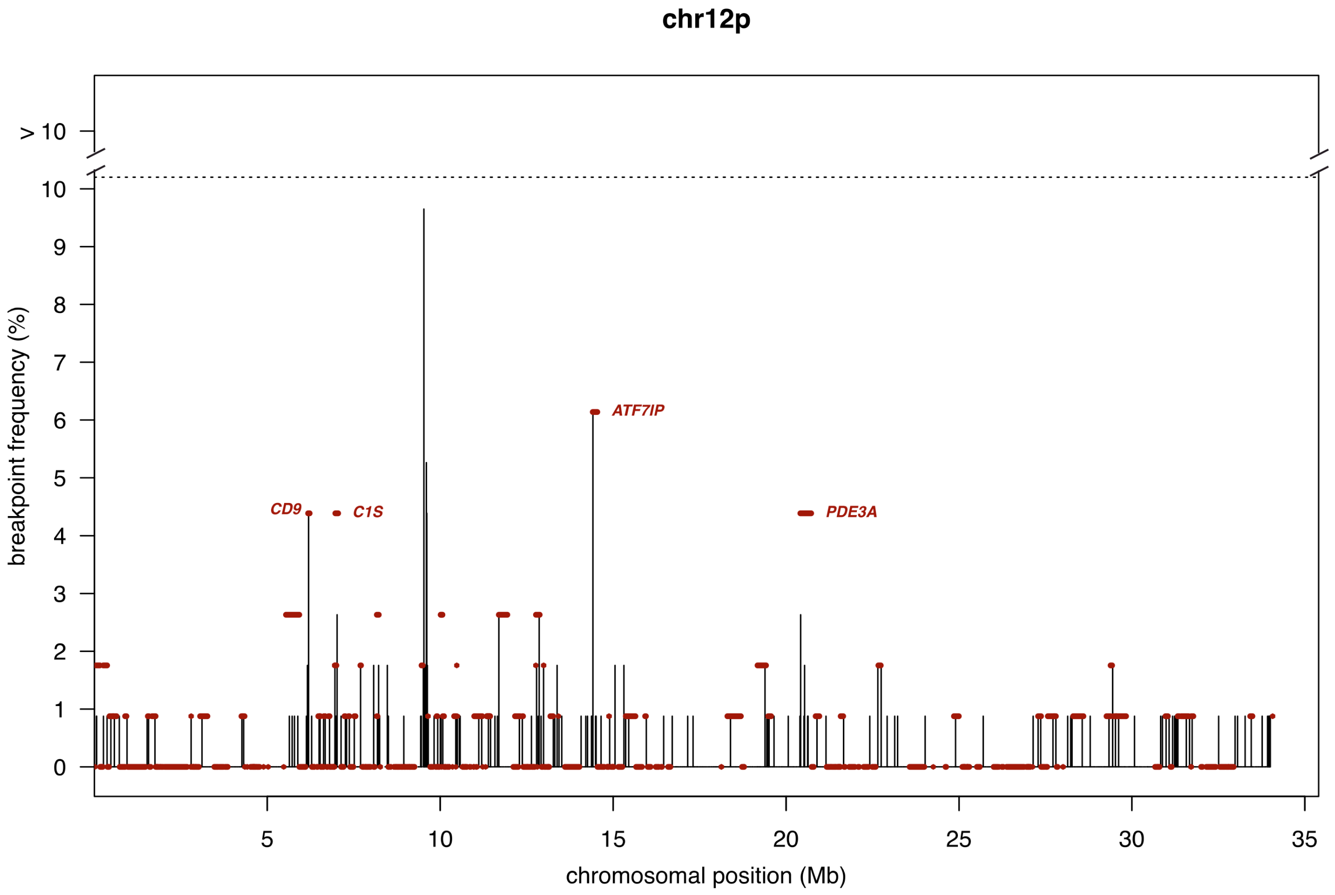
chr12p
> 10
breakpoint frequency (%)
CD9
C1S
ATF7IP
PDE3A
chromosomal position (Mb)

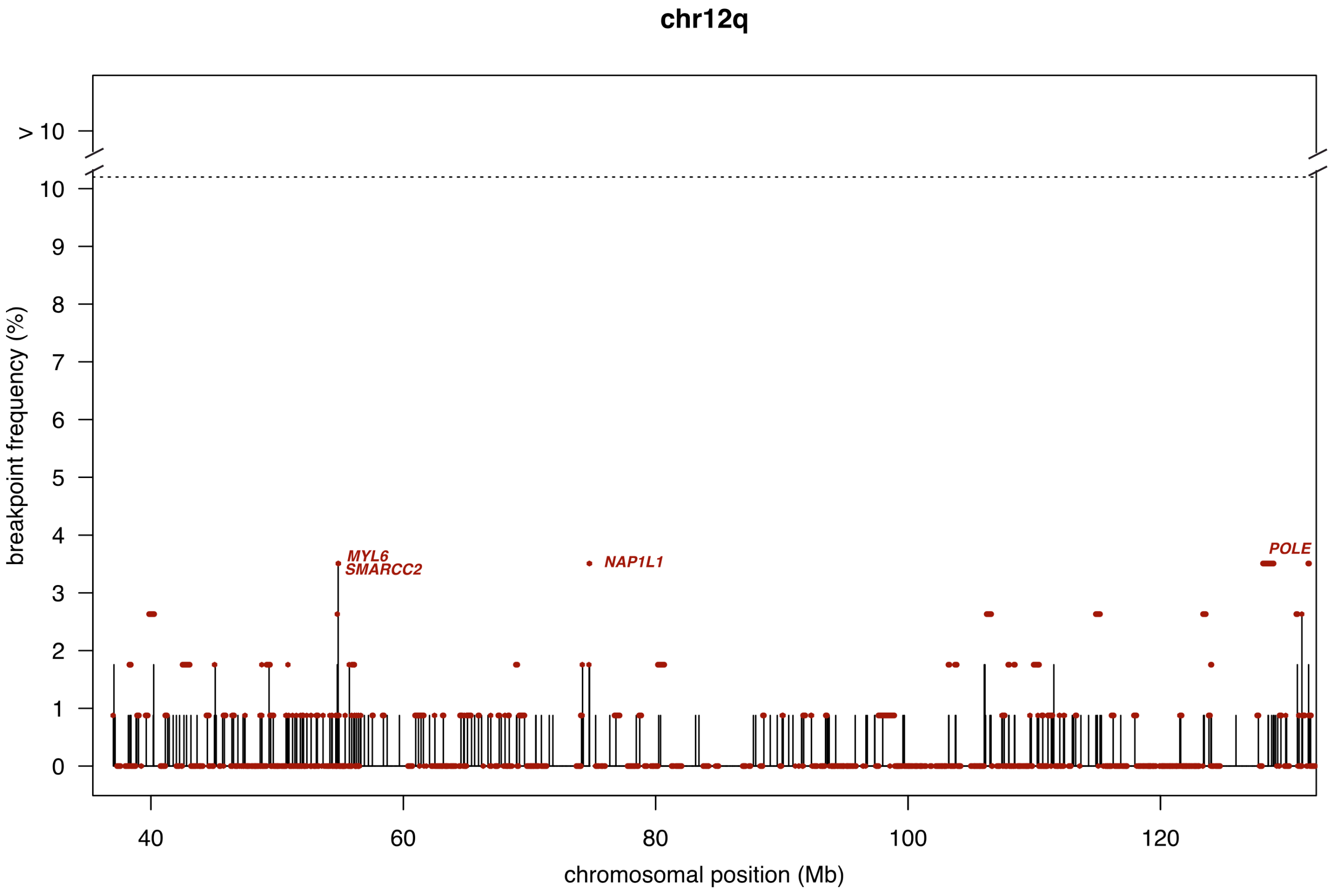
chr12q
> 10
breakpoint frequency (%)
MYL6
SMARCC2
NAP1L1
POLE
chromosomal position (Mb)

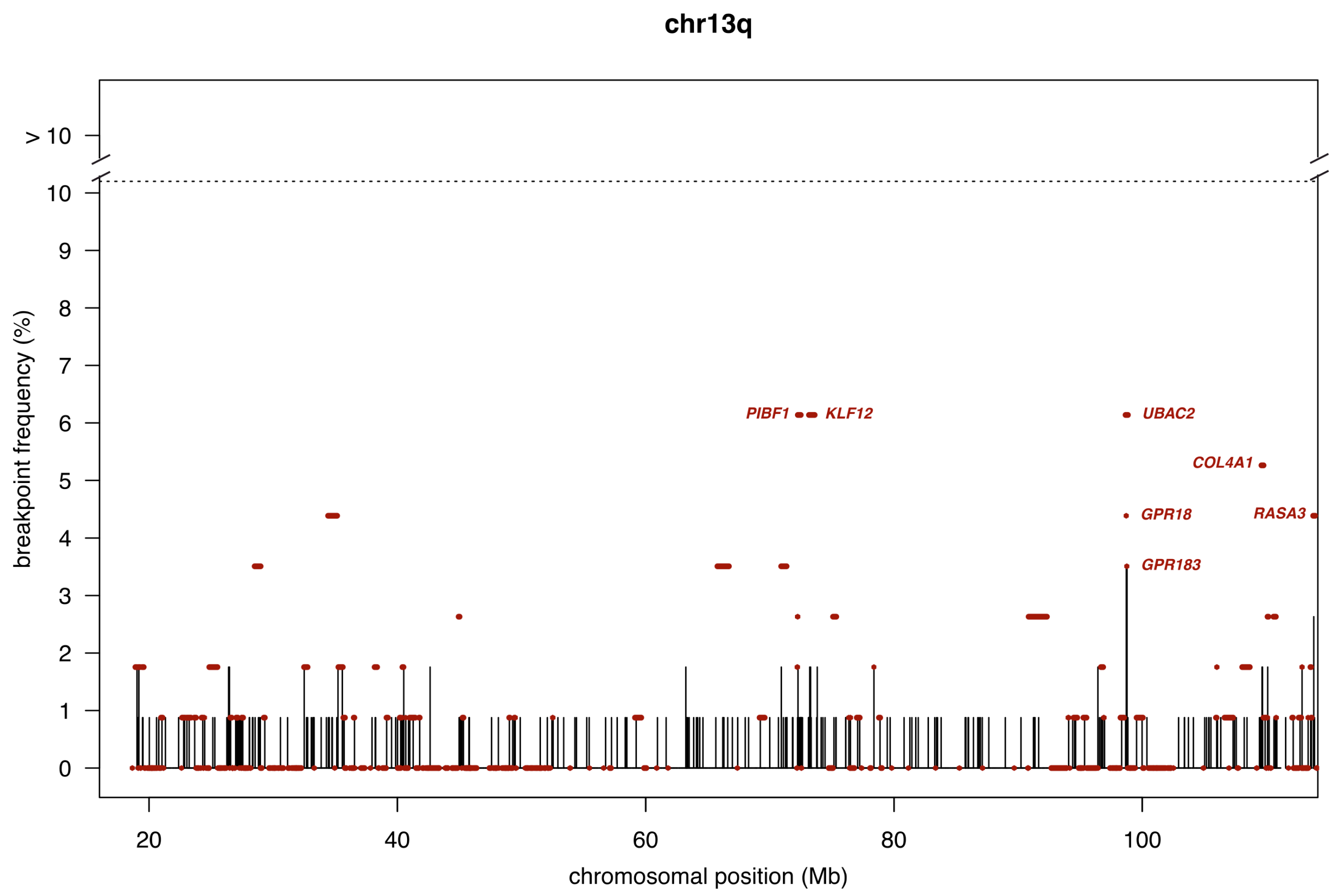
chr13q
breakpoint frequency (%)
chromosomal position (Mb)
PIBF1
KLF12
UBAC2
COL4A1
GPR18
RASA3
GPR183
> 10

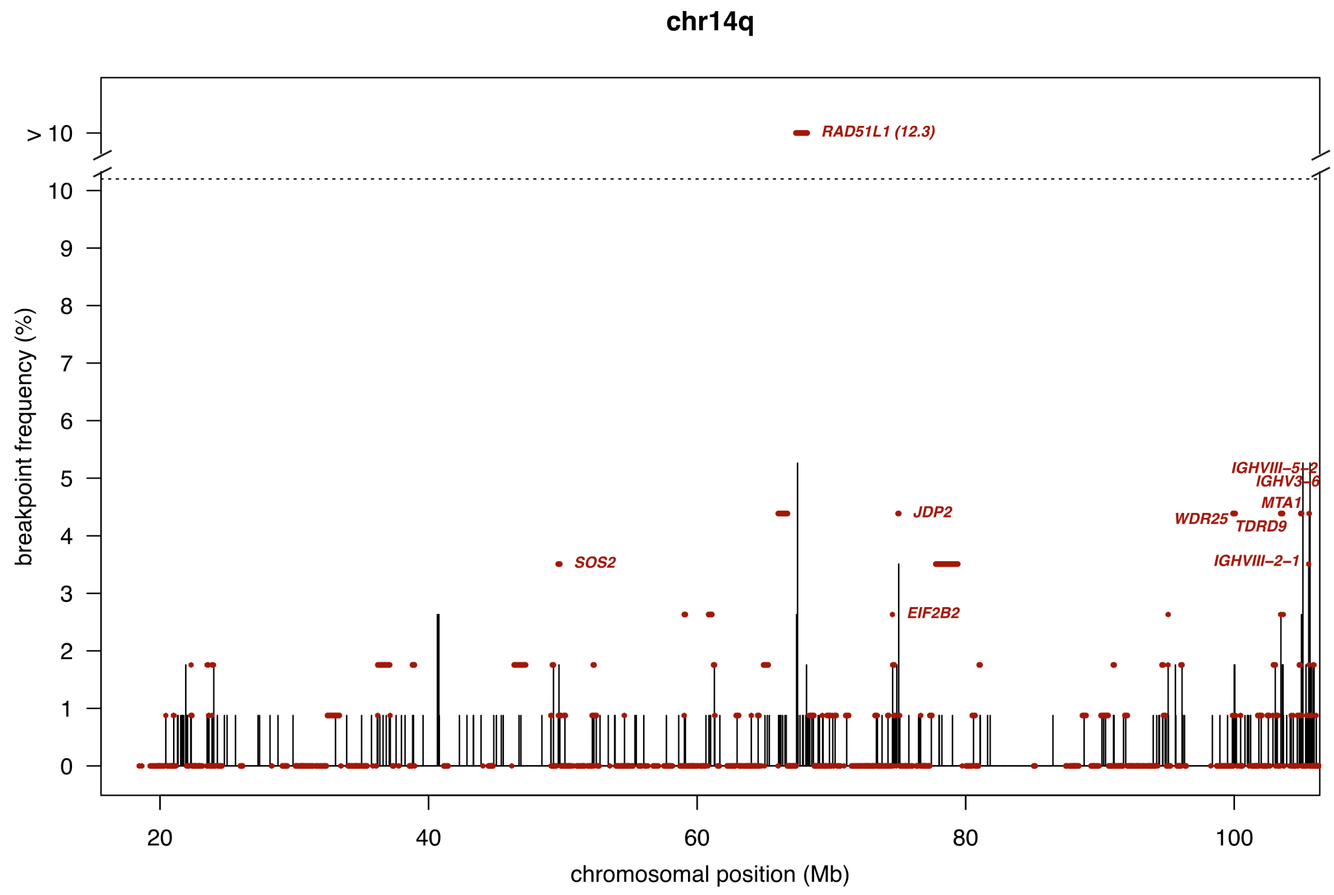
chr14q
breakpoint frequency (%)
chromosomal position (Mb)
RAD51L1 (12.3)
SOS2
JDP2
EIF2B2
IGHVIII−5−2
IGHV3−6
MTA1
WDR25
TDRD9
IGHVIII−2−1
> 10

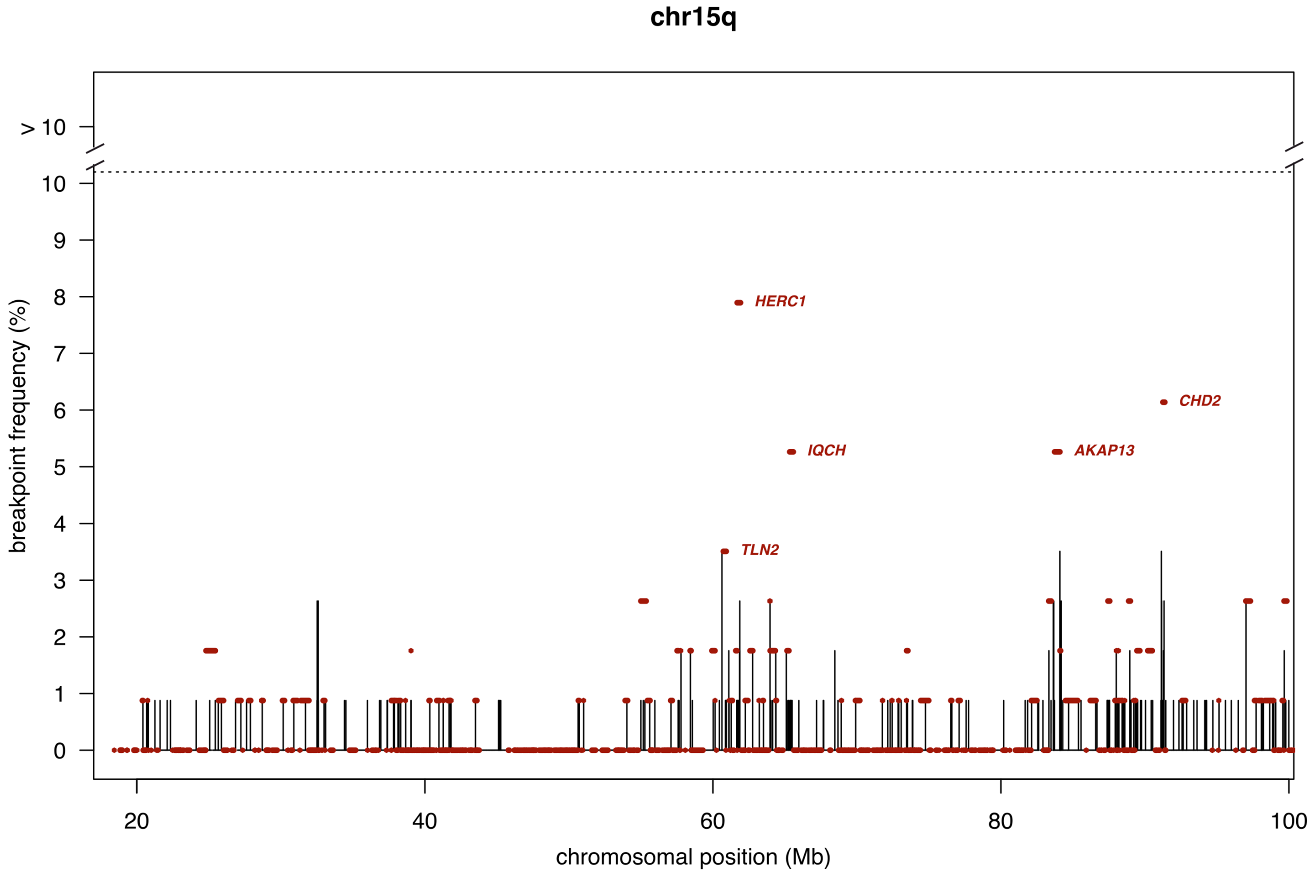
chr15q
> 10
10
9
8
7
6
5
4
3
2
1
0
breakpoint frequency (%)
HERC1
CHD2
IQCH
AKAP13
TLN2
20
40
60
80
100
chromosomal position (Mb)

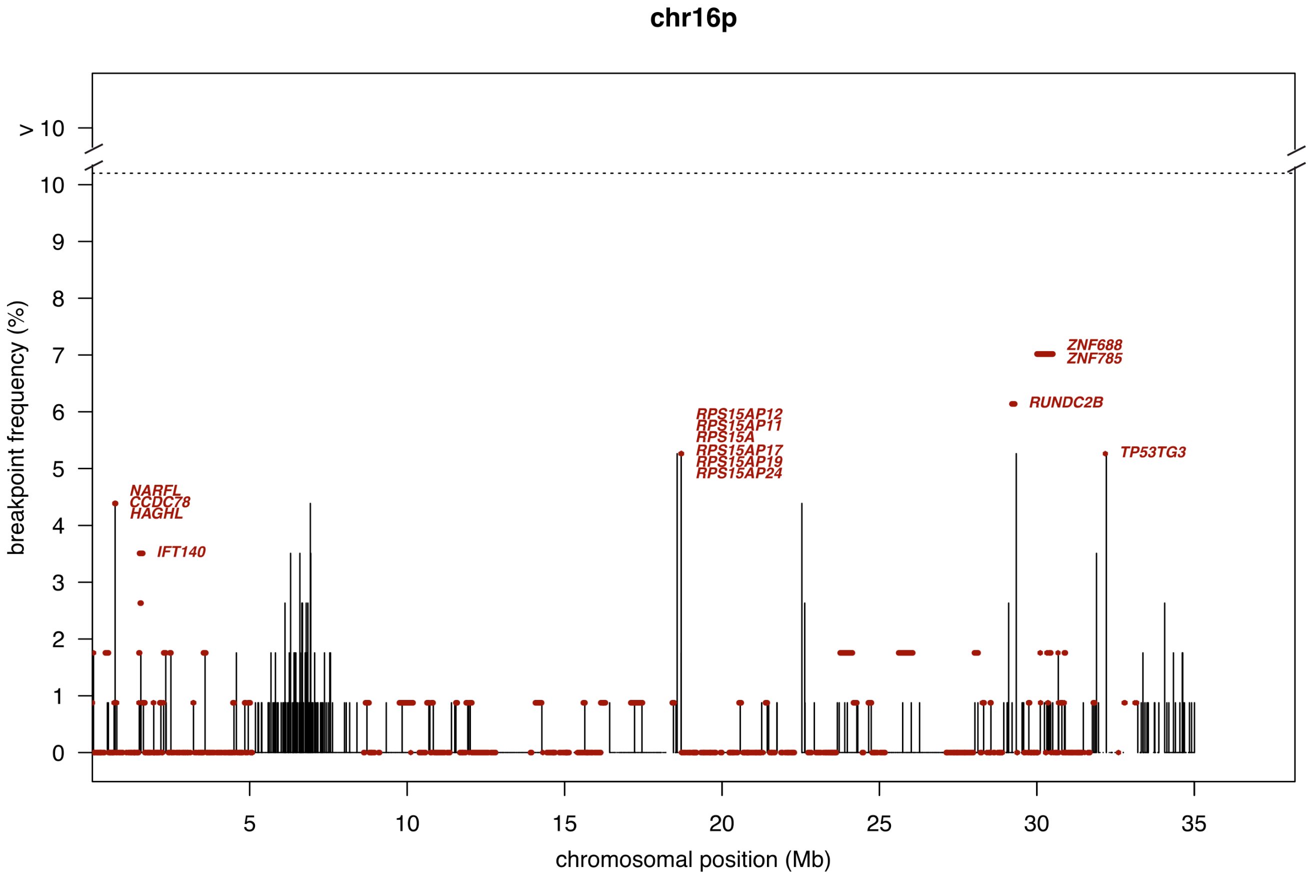
chr16p
> 10
10
9
8
7
6
5
4
3
2
1
0
breakpoint frequency (%)
ZNF688
ZNF785
RUNDC2B
RPS15AP12
RPS15AP11
RPS15A
RPS15AP17
RPS15AP19
RPS15AP24
TP53TG3
NARFL
CCDC78
HAGHL
IFT140
5
10
15
20
25
30
35
chromosomal position (Mb)

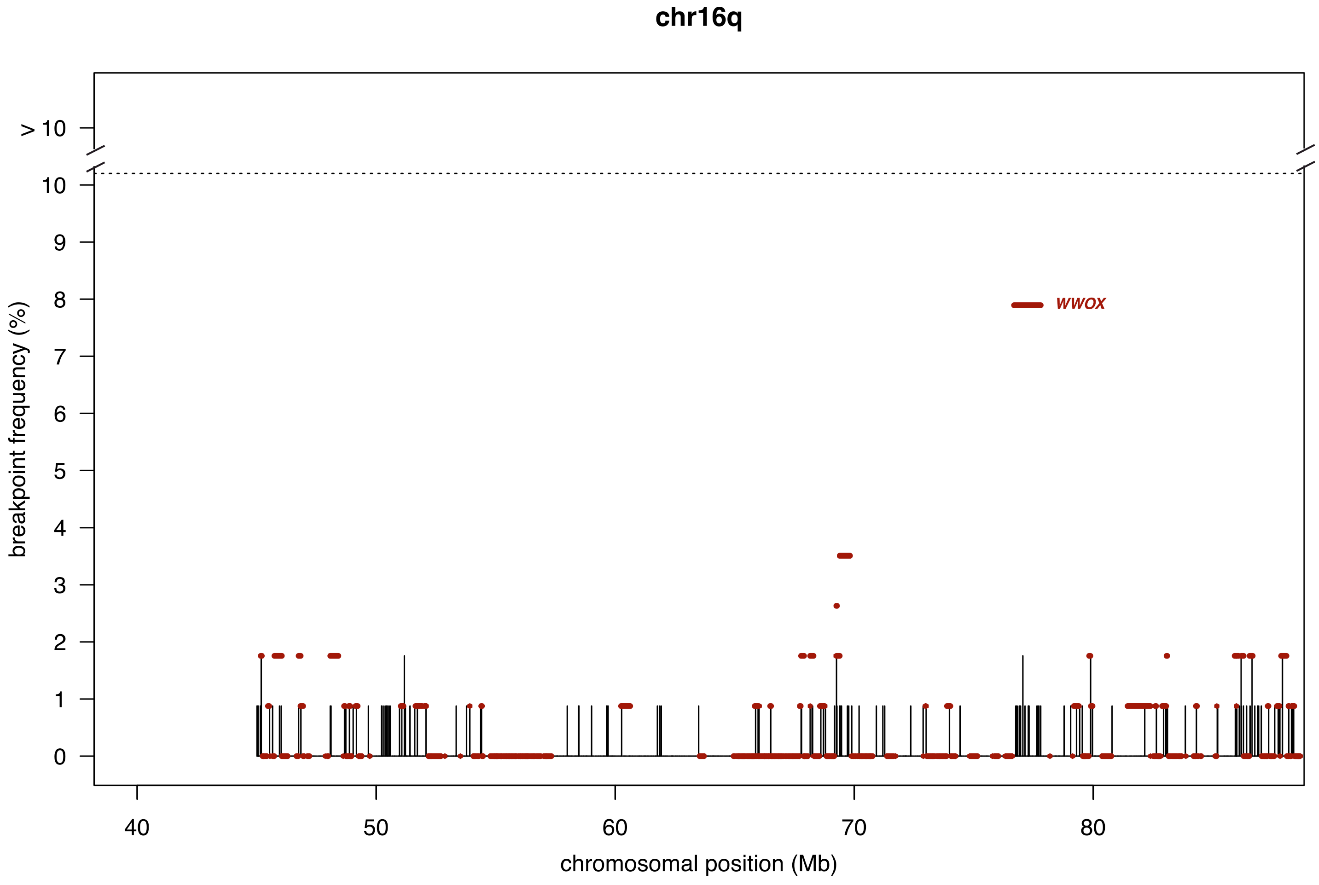
chr16q
> 10
10
9
8
7
6
5
4
3
2
1
0
breakpoint frequency (%)
WWOX
40
50
60
70
80
chromosomal position (Mb)

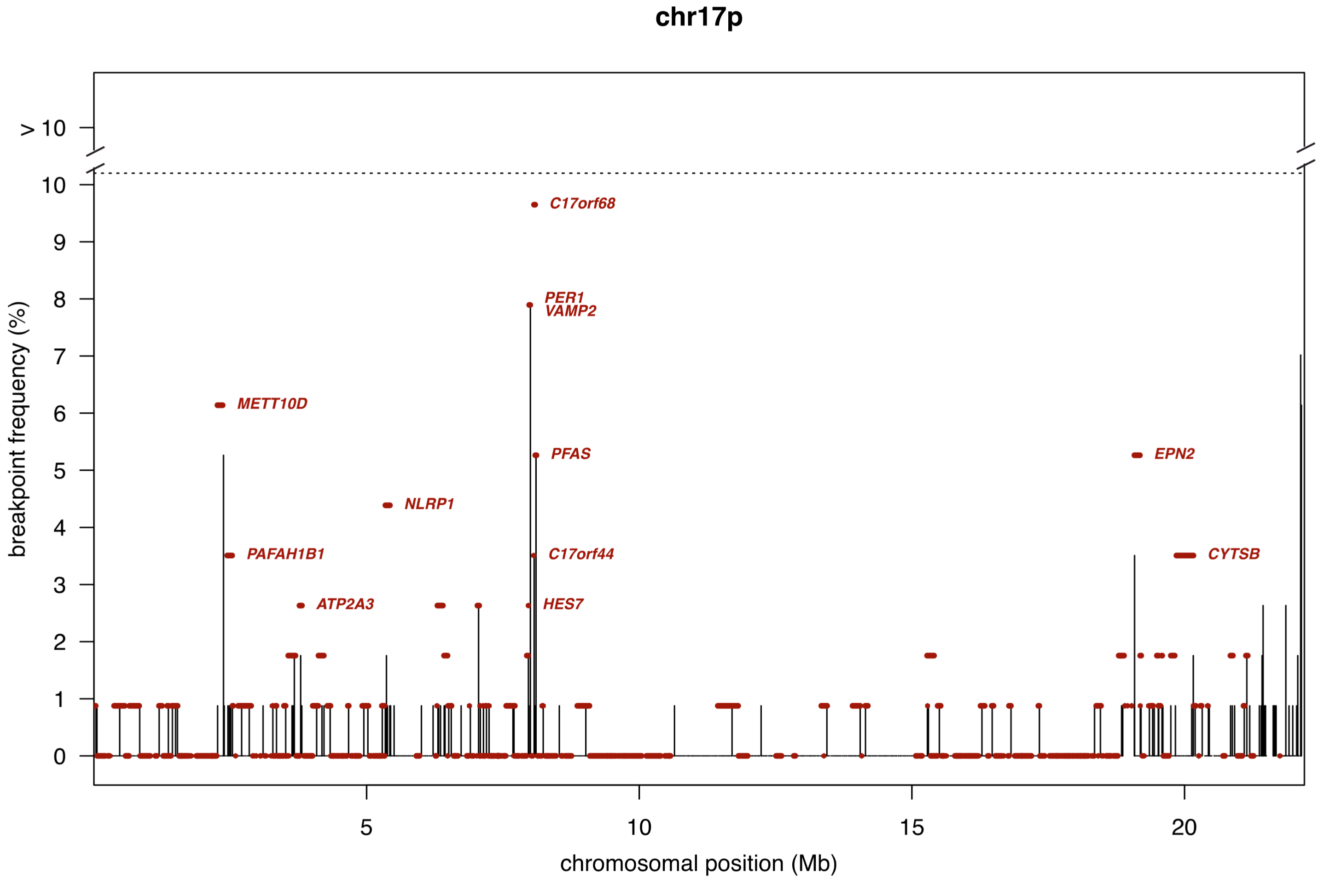
chr17p
> 10
10
9
8
7
6
5
4
3
2
1
0
breakpoint frequency (%)
C17orf68
PER1
VAMP2
METT10D
PFAS
EPN2
NLRP1
PAFAH1B1
C17orf44
CYTSB
ATP2A3
HES7
5
10
15
20
chromosomal position (Mb)

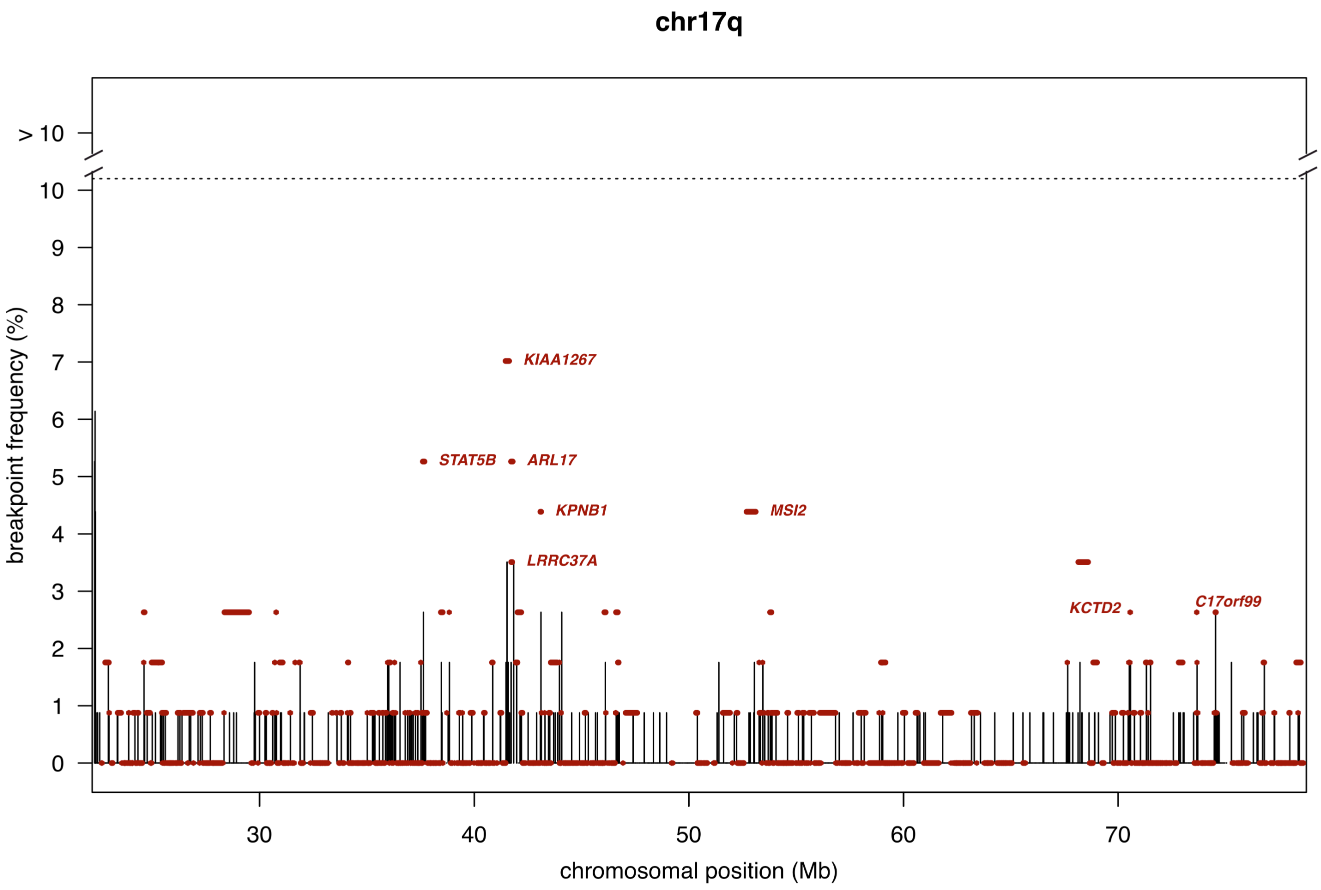
chr17q
> 10
10
9
8
7
6
5
4
3
2
1
0
breakpoint frequency (%)
KIAA1267
STAT5B
ARL17
KPNB1
MSI2
LRRC37A
KCTD2
C17orf99
30
40
50
60
70
chromosomal position (Mb)

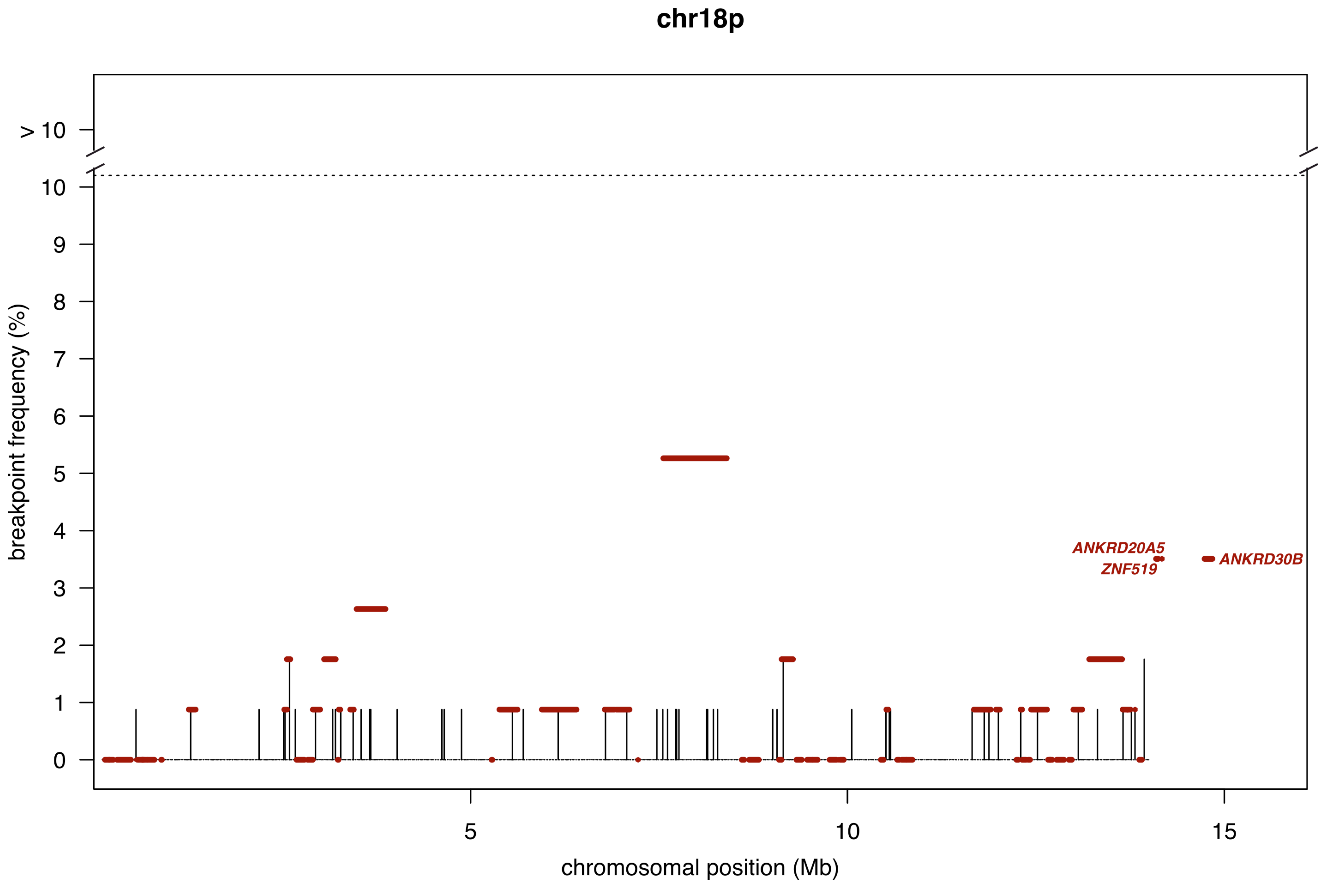
chr18p
> 10
10
9
8
7
6
5
4
3
2
1
0
breakpoint frequency (%)
ANKRD20A5
ZNF519
ANKRD30B
5
10
15
chromosomal position (Mb)

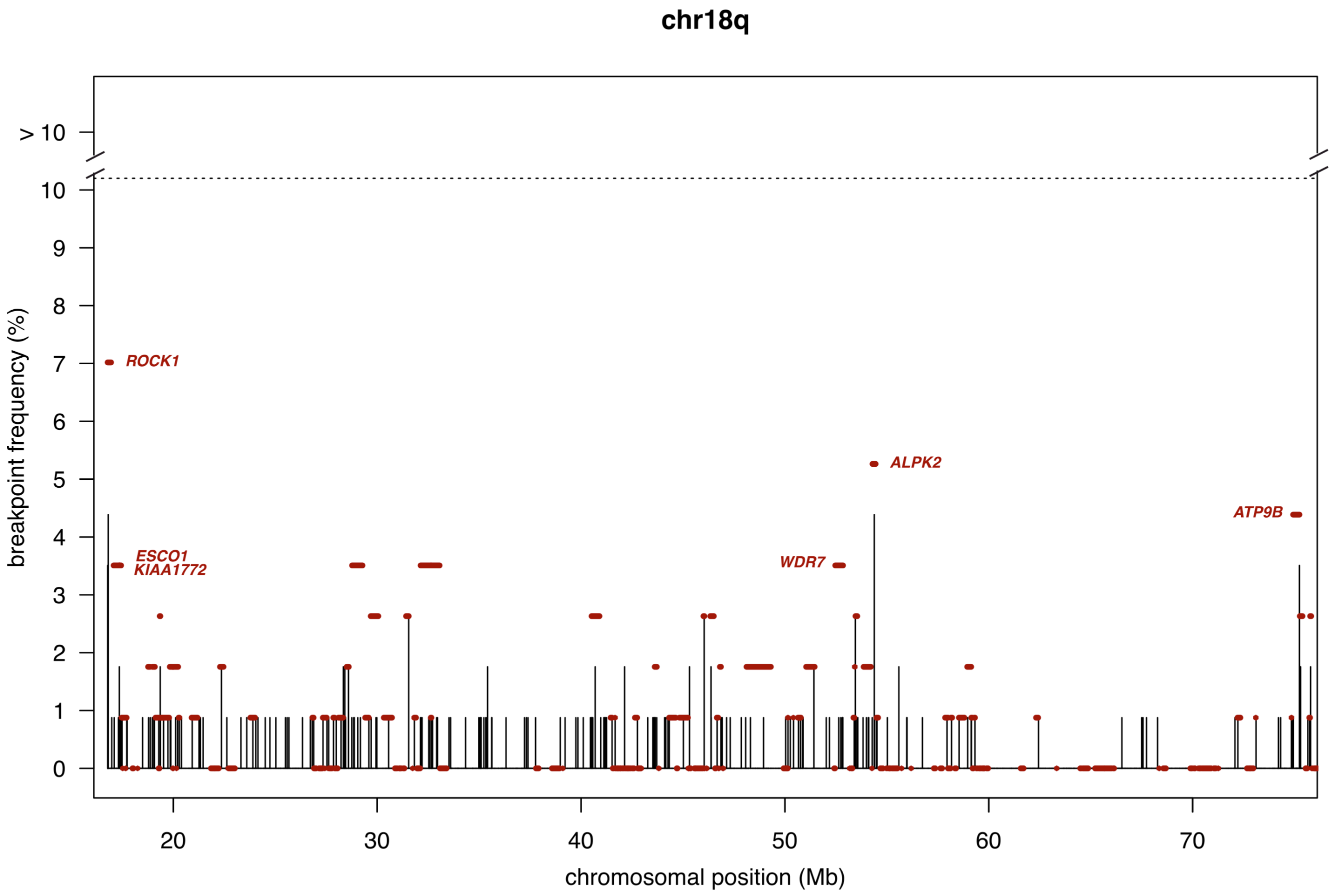

chr18q
> 10
10
9
8
7
6
5
4
3
2
1
0
breakpoint frequency (%)
ROCK1
ESCO1
KIAA1772
ALPK2
WDR7
ATP9B
20
30
40
50
60
70
chromosomal position (Mb)

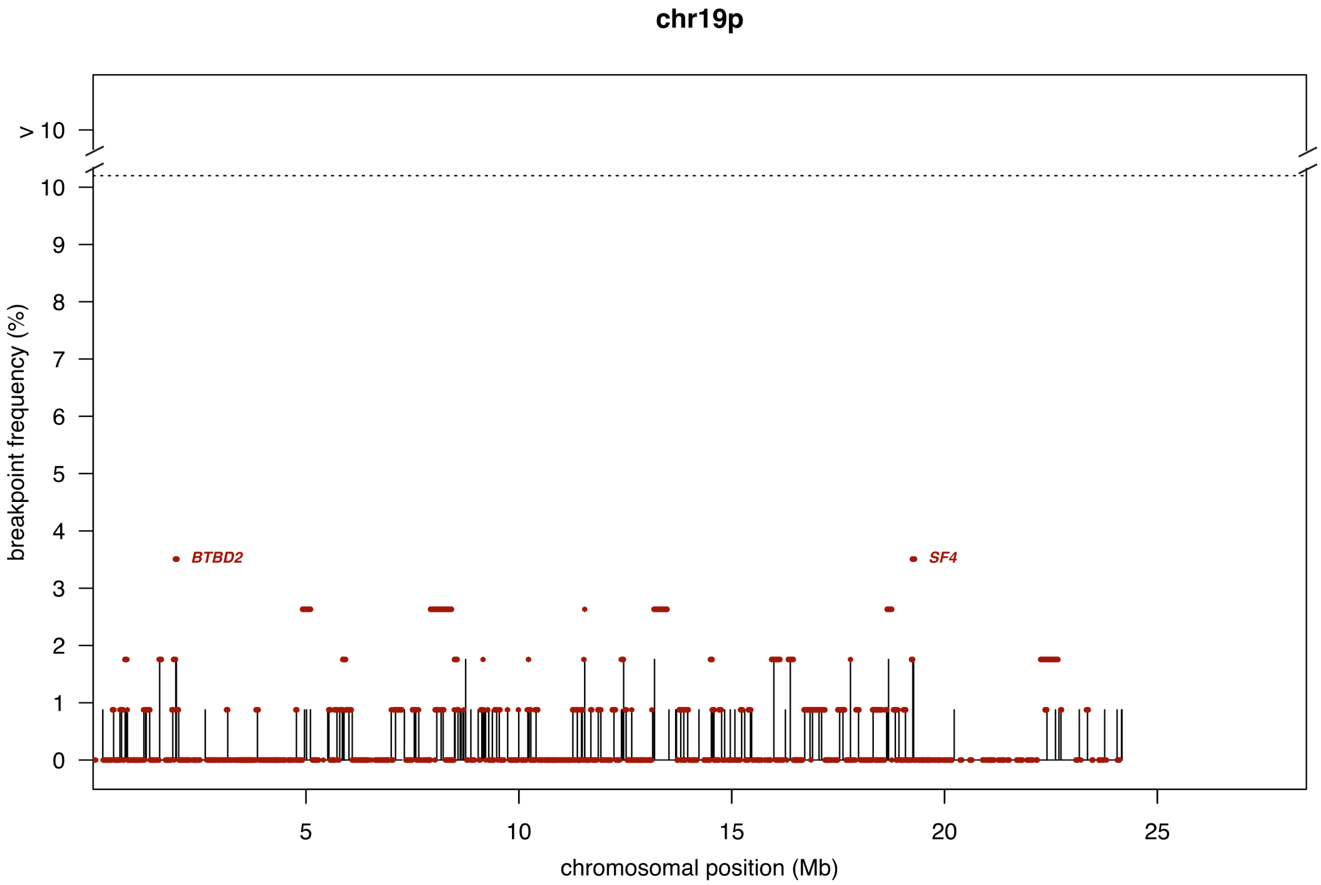

chr19p
> 10
10
9
8
7
6
5
4
3
2
1
0
breakpoint frequency (%)
BTBD2
SF4
5
10
15
20
25
chromosomal position (Mb)

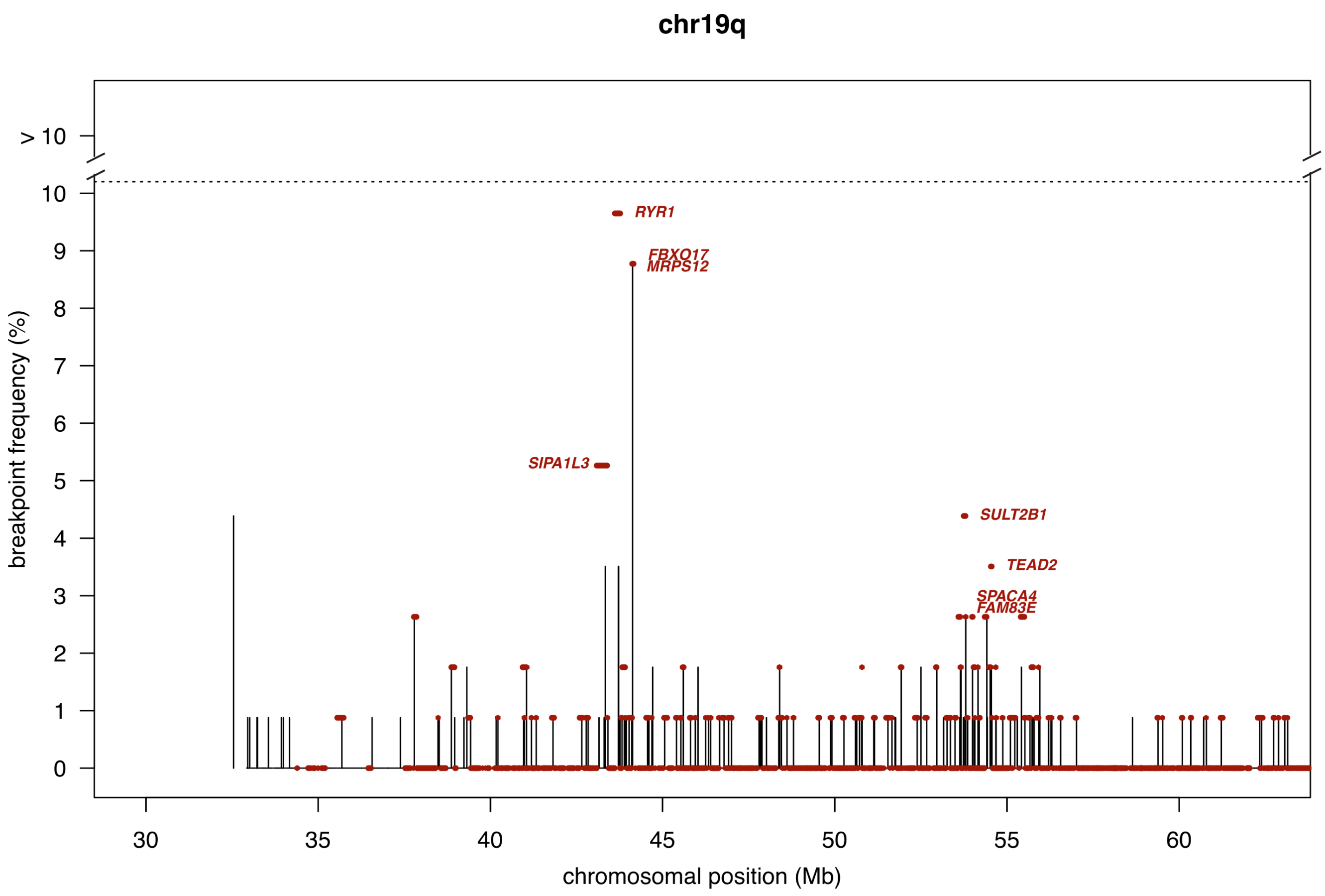

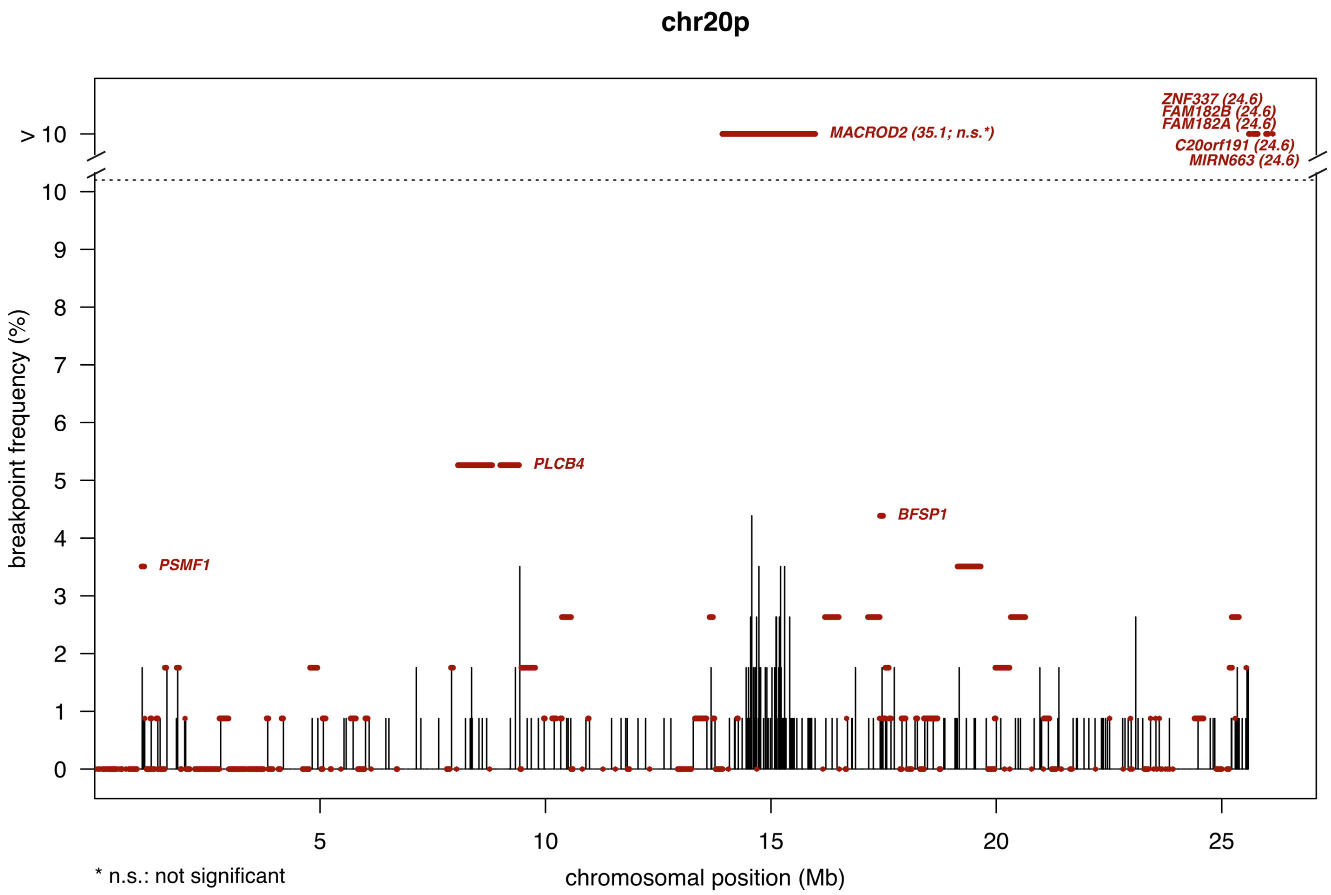

* n.s.: not significant

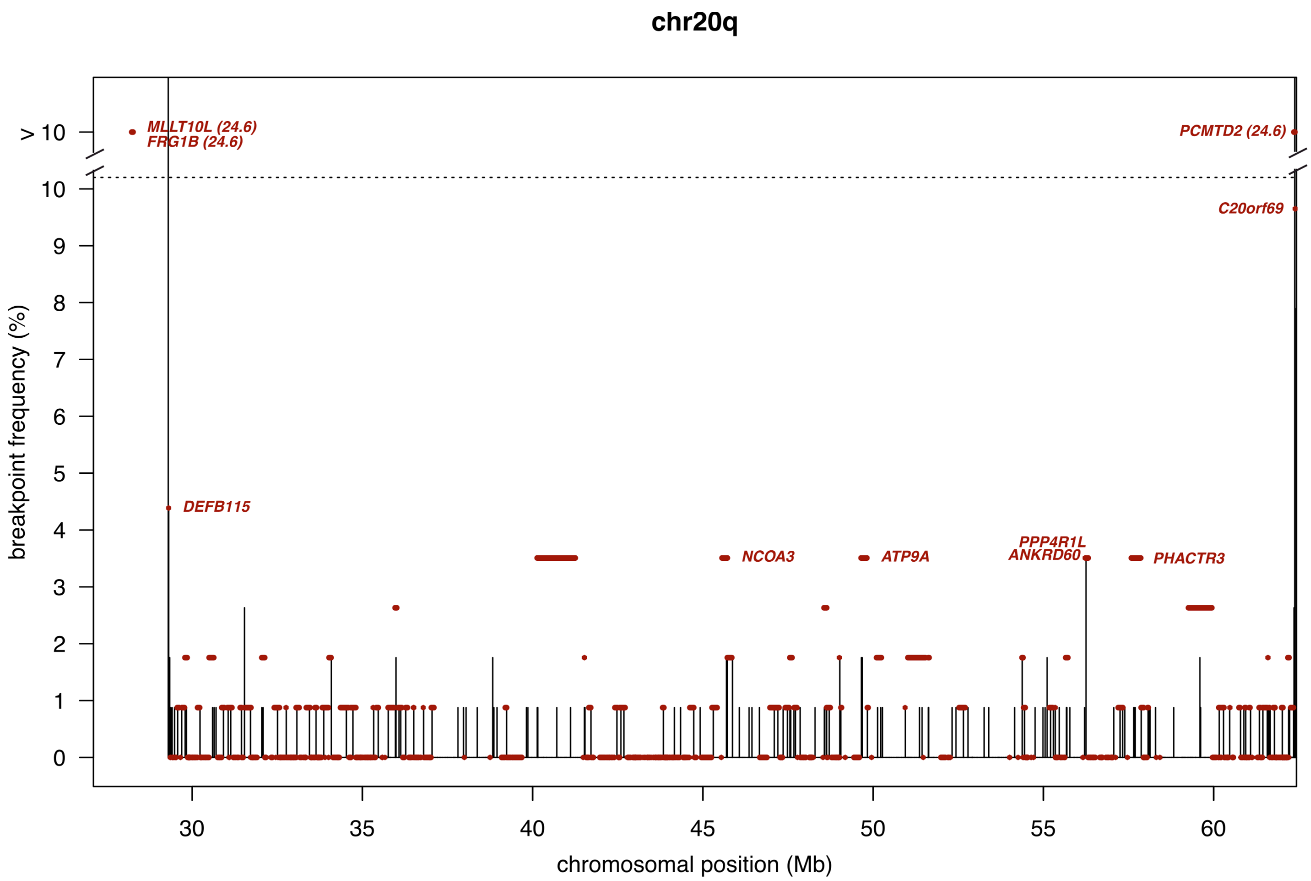
chr20q
> 10
MLLT10L (24.6)
FRG1B (24.6)
PCMTD2 (24.6)
C20orf69
DEFB115
NCOA3
ATP9A
PPP4R1L
ANKRD60
PHACTR3
breakpoint frequency (%)
chromosomal position (Mb)

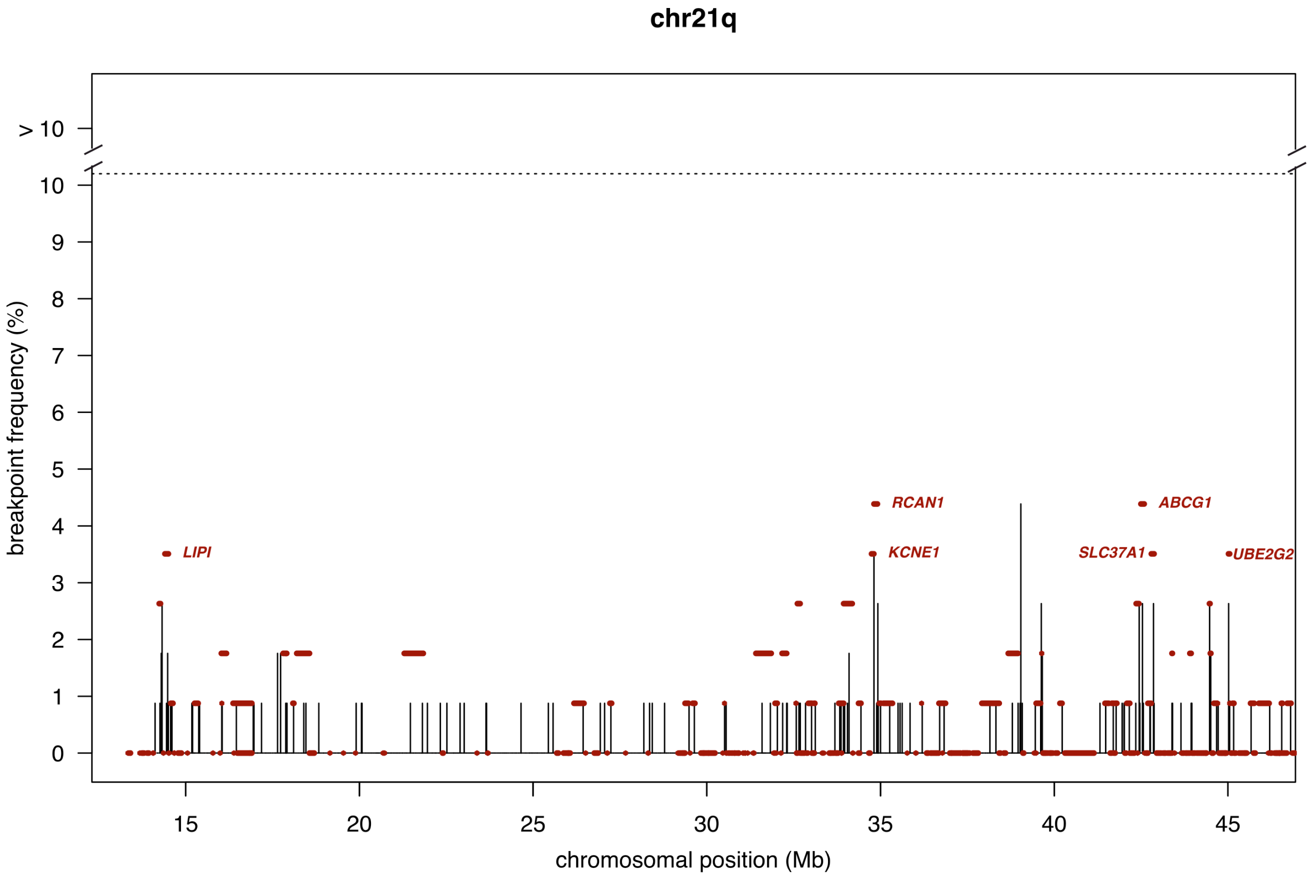
chr21q
> 10
LIPI
RCAN1
KCNE1
ABCG1
SLC37A1
UBE2G2
breakpoint frequency (%)
chromosomal position (Mb)

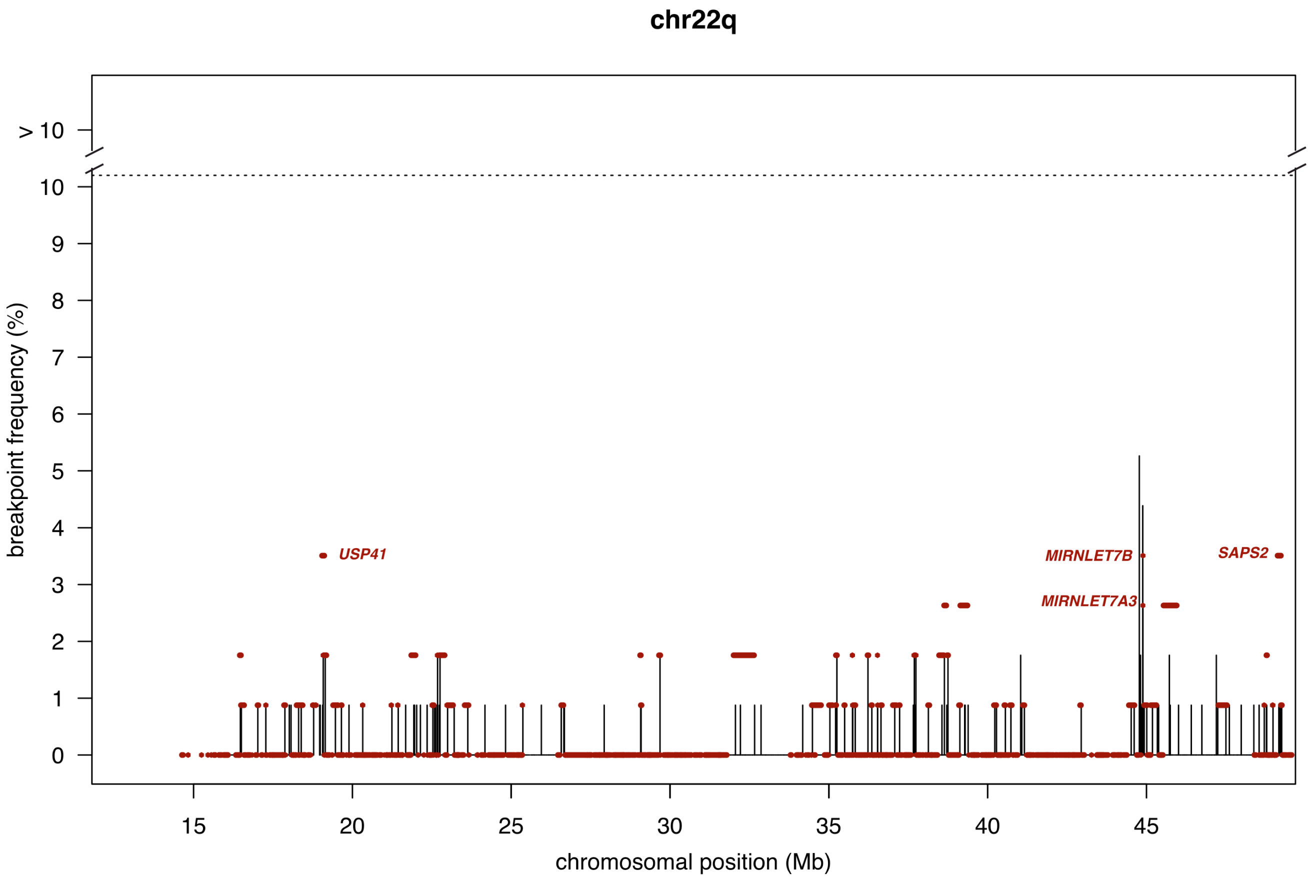

chr22q
> 10
10
9
8
7
6
5
4
3
2
1
0
breakpoint frequency (%)
USP41
MIRNLET7B
MIRNLET7A3
SAPS2
15
20
25
30
35
40
45
chromosomal position (Mb)

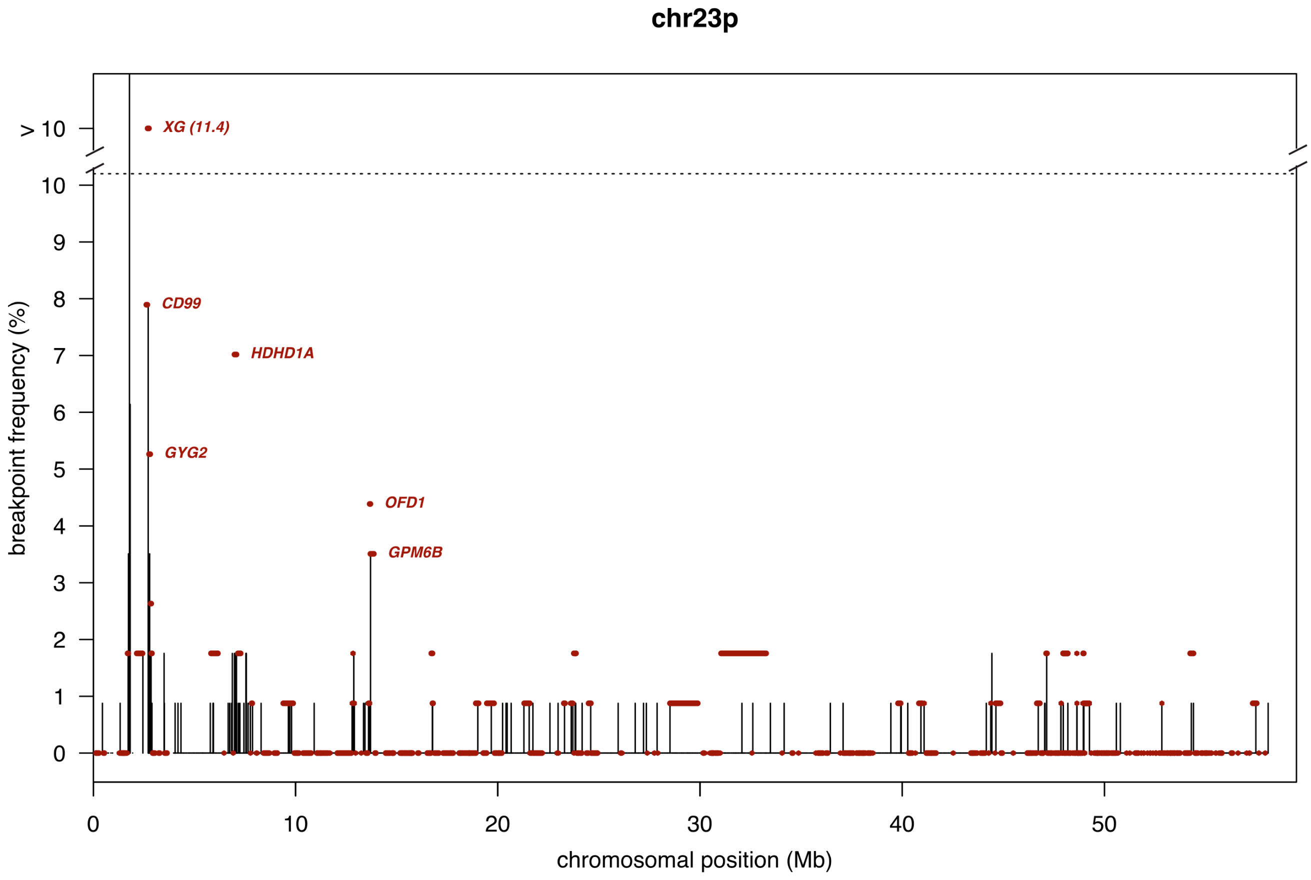

chr23p
> 10
XG (11.4)
10
9
8
7
6
5
4
3
2
1
0
breakpoint frequency (%)
CD99
HDHD1A
GYG2
OFD1
GPM6B
0
10
20
30
40
50
chromosomal position (Mb)

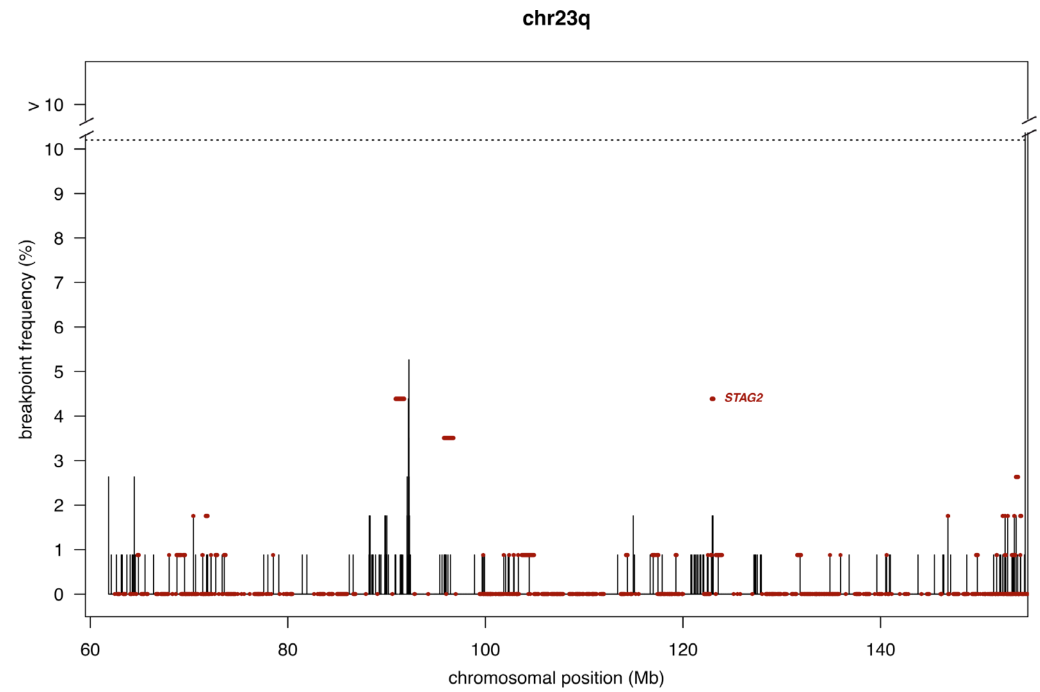

Supplementary Figure S3: Graphical representation of CNA-associated chromosomal breakpoint frequencies and their distribution over chromosomes 1-22 and X (numbered 23). The X-axes depict the genomic position in Mb. The Y-axes depict the chromosomal breakpoint frequencies across the cohort of 114 MSS stage II and III colon cancer samples. Breakpoint frequencies are indicated on array-CGH probe-level (vertical black bars) and on gene-level (horizontal red bars). Recurrent breakpoint genes (FDR < 0.1) are named. When the gene breakpoint frequency exceeded 10% (horizontal dashed line), the breakpoint frequency (%) follows the gene name.

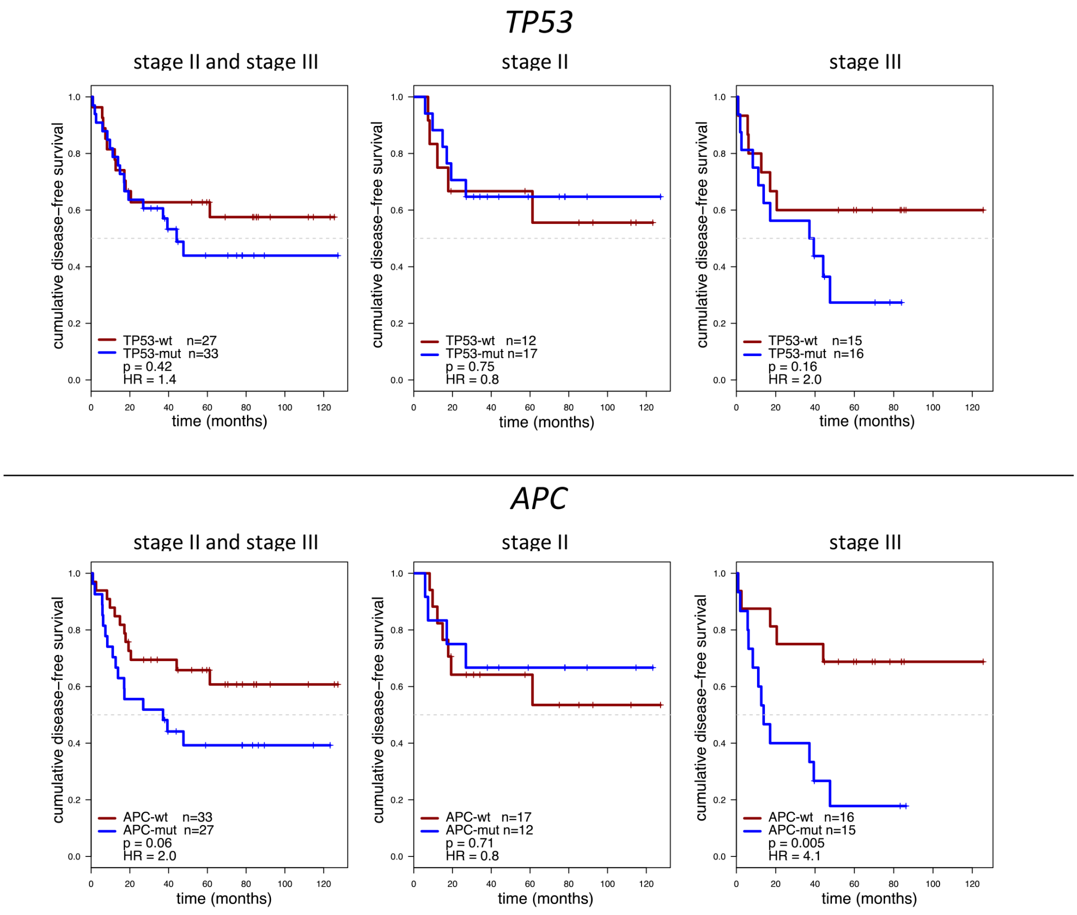

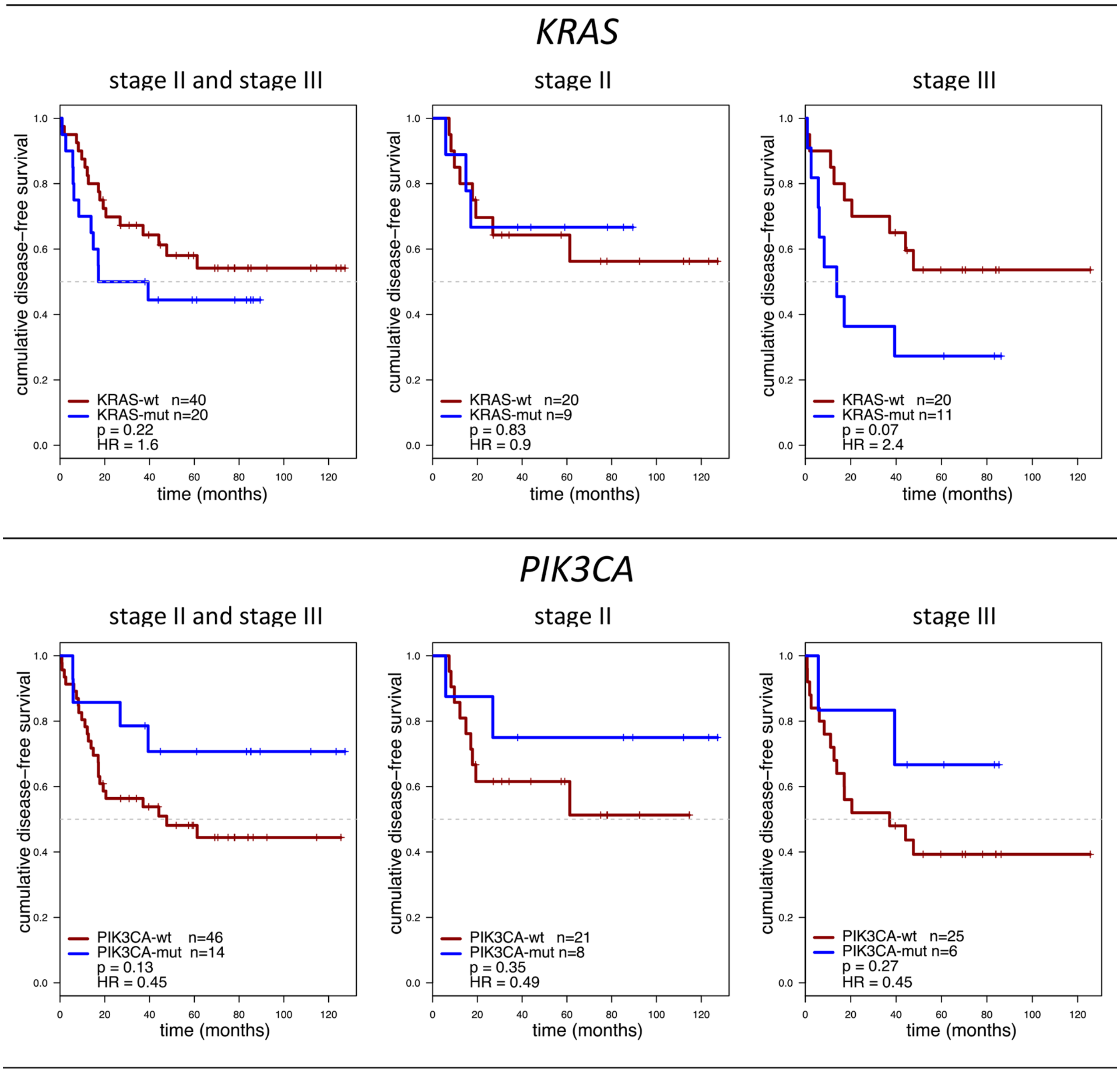

Supplementary Figure S4: Kaplan-Meier curves for disease-free survival (in months) of gene mutation status for 60 colon cancer patients and stratified for stage II (*n* = 29) and stage III (*n* = 31) in univariate analyses. Survival differences were tested by log-rank tests. The Kaplan-Meier curve is not included when the gene mutation frequency was less than 10% (*n* = 6), which was the case for the genes *FBXW7, SMAD4, BRAF and NRAS*.

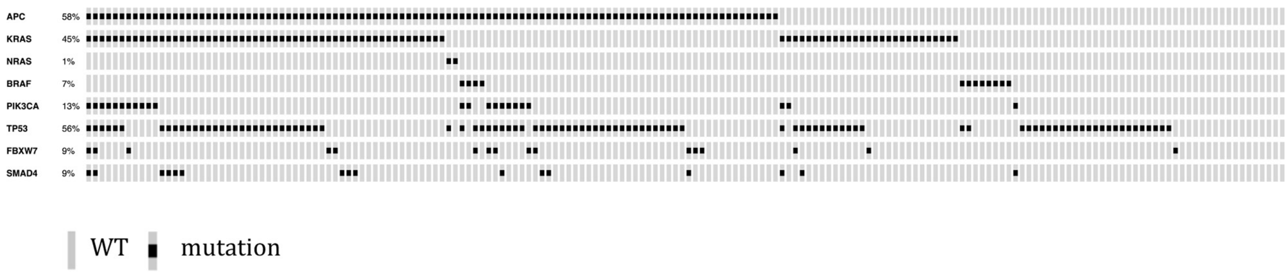

Supplementary Figure S5: Oncoprint visualizing the gene mutation status of *APC, KRAS, NRAS, BRAF, PIK3CA, TP53, FBXW7* and *SMAD4* assessed by TSACP analysis for 180 MSS advanced CRCs. The rows indicate the gene mutation status of the 180 samples (grey bars) and the black spots depict mutations.

Supplementary Table S1: Chromosomal breakpoints, probe-level. See Supplementary_ Table_S1.

Supplementary Table S2: Chromosomal breakpoints, gene-level. See Supplementary_ Table_S2.

Supplementary Table S3: Overview of samples used for CNA and TSACP analysis

Overview of clinical characteristics of samples used for CNA and TSACP analysis

		CNA analysis				TSACP analysis		
		**all (*n* = 114)**	**stage II (*n* = 57)**	**stage III (*n* = 57)**		**all (*n* = 60)**	**stage II (*n* = 29)**	**stage III (*n* = 31)**
**recurrent disease**	**No**	65 (57.0)	35 (61.4)	30 (52.6)		32 (53.3)	18 (62.1)	14 (45.2)
	**Yes**	49 (43.0)	22 (38.6)	27 (47.4)		28 (46.7)	11 (37.9)	17 (54.8)
**adjuvant therapy**	**No**	56 (49.1)	55 (96.5)	1 (1.8)		29 (48.3)	29 (100.0)	0 (0.0)
	**Yes***	58 (50.9)	2 (3.5)	56 (98.2)		31 (51.7)	0 (0.0)	31 (100.0)

Values in parentheses are percentages.
*Adjuvant chemotherapy: 5-fluorouracil and leucovorin (5-FU/LV) mono therapy.

Supplementary Table S4: CNA-regions and associations with disease recurrence or stage. See Supplementary_ Table_S4.

Supplementary Table S5: Pools of genes that share probe(s) associated with chromosomal breakpoints

Gene pools	Genes (Ensembl54)
Pool_1	*HSD17B7P2 & ZNF33A & ZNF33B & ZNF37A & ZNF37B*
Pool_2	*ANXA8L1 & CTSLL7 & FAM25B & FAM25C & FAM25G & FAM35B2 & GLUDP8*
Pool_3	*IGHV3-6 & IGHVIII-5-2*
Pool_4	*C20orf191 & FAM182A & FAM182B & FRG1B & MIRN663 & MLLT10L & ZNF337*
Pool_5	*ARL17 & LRRC37A*
Pool_6	*DEFB105B & DEFB106B & DEFB107B*
Pool_7	*CD99 & XG*
Pool_8	*ANKRD20A5 & ANKRD30B & ZNF519*
Pool_9	*CCDC78 & HAGHL & NARFL*
Pool_10	*AMACR & C1QTNF3*
Pool_11	*GPM6B & OFD1*
Pool_12	*KCNE1 & RCAN1*
Pool_13	*EMB & PARP8*
Pool_14	*HIST1H1A & HIST1H3A*
Pool_15	*HOXA1 & HOXA2*
Pool_16	*HOXA10 & HOXA11*
Pool_17	*PER1 & VAMP2*
Pool_18	*HIST1H2BO & HIST1H3H & OR2B2*
Pool_19	*C17orf44 & C17orf68 & PFAS*
Pool_20	*RPS15A & RPS15AP11 & RPS15AP12 & RPS15AP17 & RPS15AP19 & RPS15AP24*
Pool_21	*GPR18 & GPR183 & UBAC2*
Pool_22	*CBWD3 & FOXD4L3 & PGM5*
Pool_23	*EPCAM & MSH2*
Pool_24	*ZNF688 & ZNF785*
Pool_25	*MYL6 & SMARCC2*
Pool_26	*ANKRD60 & PPP4R1L*
Pool_27	*CDX1 & SLC6A7*
Pool_28	*C20orf69 & PCMTD2*
Pool_29	*FBXO17 & MRPS12*
Pool_30	*PACRG & PARK2*
Pool_31	*PPP1R12B & SYT2*

Genes grouped in "pools" that share same (breakpoint) array-CGH probes.

Supplementary Table S6: Gene mutation matrix. See Supplementary_ Table_S6.

Supplementary Table S7: Gene mutation frequencies

	Overall (n = 60)		Stage II (n = 29)		Stage III (n = 31)	
**Gene**	**Number of samples with gene mutation**	**Mutation frequency (%)**	**Number of samples with gene mutation**	**Mutation frequency (%)**	**Number of samples with gene mutation**	**Mutation frequency (%)**
TP53	33	55.0%	17	58.6%	16	51.6%
APC	27	45.0%	12	41.4%	15	48.4%
KRAS	20	33.3%	9	31.0%	11	35.5%
PIK3CA	14	23.3%	8	27.6%	6	19.4%
FBXW7	5	8.3%	3	10.3%	2	6.5%
BRAF	4	6.7%	1	3.4%	3	9.7%
SMAD4	3	5.0%	3	10.3%	0	0.0%
NRAS	2	3.3%	1	3.4%	1	3.2%

Gene mutations frequencies in 60 stage II and III colon cancer samples.
